# Investigating the Chemical–Biological Link in *Ziziphora galinae* Extracts for the Discovery of Novel Raw Materials via *In Silico* and In Vitro Assays

**DOI:** 10.1002/fsn3.70875

**Published:** 2025-09-01

**Authors:** Serdar Korpayev, Mehmet Veysi Cetiz, Jasmina Glamočlija, Neda Aničić, Uroš Gašić, Dejan Stojković, Mirap Agamuradov, Hemra Hamrayev, Stefano Dall'Acqua, Gokhan Zengin

**Affiliations:** ^1^ Biotechnology Institute Ankara University Ankara Turkey; ^2^ Department of Medical Biochemistry, Faculty of Medicine Harran University Sanliurfa Turkey; ^3^ Department of Plant Physiology, Institute for Biological Research “Siniša Stanković” – National Institute of Republic of Serbia University of Belgrade Belgrade Serbia; ^4^ Saint Petersburg State Pediatric Medical University St Petersburg Russia; ^5^ Department of Chemical and Petroleum Engineering University of Calgary Calgary Alberta Canada; ^6^ Department of Pharmaceutical and Pharmacological Sciences University of Padova Padua Italy; ^7^ Department of Biology, Science Faculty Selcuk University Konya Turkey

**Keywords:** antimicrobial activity, antioxidants, food borne bacteria, MM‐PBSA, molecular docking, phytochemicals, *Ziziphora galinae*

## Abstract

This study examined the impact of the extraction methods (70% ethanolic extraction, infusion) on the overall biological profile and concentration of phenolic compounds of *Ziziphora galinae (ZG)*. Infusion yielded significantly higher phenolics and flavonoids (75.73 ± 0.22 mg GAE/g and 8.87 ± 0.36 mg RE/g) than the ethanol extract (10.44 ± 0.1 and 2.44 ± 0.17, respectively). Nineteen key bioactive compounds, including caffeoylquinic acids, rutin, and p‐coumaric acid, were identified. Both extracts exhibited strong antibacterial and moderate antifungal activity, but no notable cytotoxicity (IC50 > 400 μg/mL). Furthermore, *in silico* analyses involving molecular docking, molecular dynamics (MD) simulations, and MM‐PBSA free energy calculations revealed that the phytochemicals identified from ZG exhibited strong binding affinities and high structural stability against key human metabolic enzymes and essential bacterial proteins involved in cell wall biosynthesis and DNA replication. Based on these results, the varying effects produced by the two extracts of endemic ZG may be attributed to the presence of distinct compounds, making them a valuable source of bioactive compounds to benefit human health.

## Graphical abstract

*Ziziphora galinae* may be attributed to the presence of distinct compounds, making them a valuable source of bioactive compounds to benefit human health.

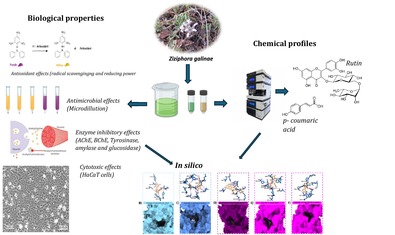

## 1 Introduction

The genus *Ziziphora* L. (Lamiaceae) comprises roughly 30 species of annual and perennial herbs and subshrubs, extensively spread across Southern and Eastern Europe, Central Asia, and the Middle East. These plants have long been used in traditional medicine, especially in Turkmen, Turkish, Kazakh, and Iranian ethnobotanical techniques, and are well known for their aromatic qualities (Ilhan et al. 2025; Šmejkal et al. 2016). *Ziziphora* species are widely used in traditional medicine across many cultures because of their rich phytochemical composition, which includes flavonoids, triterpenes, and monoterpenic essential oils (Mohammadhosseini 2017; Selvi and Satil 2020; Zhaparkulova et al. 2022). Among these, the relatively endemic *Ziziphora galinae* has attracted interest lately because of its possible phytochemical diversity and medicinal significance.

*Ziziphora galinae* (ZG) is a perennial herb that is native to Turkmenistan and belongs to the Lamiaceae family, has attracted a lot of interest because of its therapeutic qualities and historical applications in herbal medicine (Berdimuhamedow 2010; Plant List 2024). Turkmen folk medicine has a long history of using ZG, popularly known as Galina oregano. ZG is a native of many parts of Turkmenistan, especially the dry and mountainous regions (Nikitin and Gel'dikhanov 1988). ZG is a little, aromatic plant that reaches a height of 10 to 20 cm. Its tiny leaves have a fuzzy appearance due to the thin hairs covering them. The summertime blooms of ZG are usually pink or purple in hue. Based on genus‐level flowering patterns and essential oil content, ZG is best harvested in late spring to early summer (May–July), when its aerial portions exhibit the highest levels of phytochemical activity. It has long been utilized for its antioxidant, antibacterial, and anti‐inflammatory qualities locally. Its biological actions are attributed to the abundance of flavonoids, essential oils, and other phytochemicals it contains (Šmejkal et al. 2016). Other *Ziziphora* species, such as *Z. clinopodioides* and *Z. bungeana* (Zhaparkulova et al. 2022), on the other hand, have wider geographic ranges that include Asia, North‐West Africa, and Eastern Europe (Salehi et al. 2005; Shahbazi 2017; Shomali 2019).

The plant has traditionally been used as a natural wound healing therapy as well as a treatment for respiratory and digestive issues (Berdimuhamedow 2010). Growing interest in its possible medicinal benefits has resulted from scientific investigations in recent years, particularly by the Ministry of Health in Turkmenistan. Since diverse phytochemicals, including caffeic acid derivatives, fatty acids, flavonoids, triterpenes, sterols, pulegone, and other monoterpenoids, are considered key contributors to the pharmacological effects of *Ziziphora* species (Konyalιoglu et al. 2006; Mohammadhosseini 2017; Zhaparkulova et al. 2022), the endemic ZG holds significant promise from both pharmacognosy and phytotherapy perspectives. With an emphasis on ZG's bioactive chemicals and therapeutic potential in contemporary medicine, this paper attempts to provide a thorough overview of the plant's chemical makeup, pharmacological activity, and possible uses. To the best of our knowledge, TPC, TFC, antioxidant, and enzyme inhibition activities of different ZG extracts have never been evaluated and compared. As a result, the current study was designed to assess the antioxidant, α‐glucosidase, and α‐amylase inhibitory properties of various crudes and fractions derived from the ZG. Furthermore, to gain molecular‐level insights into the interaction mechanisms of bioactive compounds from ZG with key metabolic enzymes, molecular docking, MD simulations, and MM/PBSA (Molecular Mechanics Poisson‐Boltzmann Surface Area) free energy calculations were employed. These in silico approaches provide complementary evidence to experimental results by revealing binding affinity, stability, and interaction profiles of potential inhibitors within protein active sites.

## 2 Materials and Methods

### 2.1 Study Material

Endemic *Ziziphora galinae* specimens were collected from the Southwest Kopetdagh region Akhal, Turkmenistan (coordinates: 38.227875, 57.553294) during their peak flowering period (late May to June 2022) to ensure maximum bioactive compound content. Plant identification was verified by the National Institute of Deserts, Flora, and Fauna of Turkmenistan. The aerial parts of the plant were air‐dried and ground into a fine powder using a laboratory mill.

### 2.2 Extraction of Materials

To prepare the infusion‐based extract of ZG, 10 g of powdered material was steeped in 200 mL of distilled hot water for 15 min. Subsequently, the mixture was filtered using a Whatman No. 1 filter and then lyophilized. In addition, 15 g of powdered aboveground ZG plant material was extracted with ethanol/water (70:30, v/v) at room temperature while stirring until the solvent turned colorless (Belwal et al. 2018). Due to limited solvent availability in the local setting, 70% ethanol was used, as it provided superior extraction efficiency. The resulting extract was filtered through Whatman filter paper, and the ethanol (70%) was evaporated at 40°C using a rotary evaporator.

### 2.3 Assay for Total Phenolics and Flavonoid

The Folin–Ciocalteu (FC) reagent method was used to evaluate the total bioactive content in different extracts of endemic ZG (Slinkard and Singleton 1977). The total phenolic content (TPC) was quantified by expressing the results as milligrams of gallic acid equivalent per gram of extract (mg GAE/g). Likewise, the total flavonoid content (TFC) was measured in terms of rutin equivalent per gram of extract (mg RE/g) (Quettier‐Deleu et al. 2000).

### 2.4 UHPLC–MS/MS Analysis of Polyphenolic Compounds

UHPLC–MS/MS analysis was conducted using a Dionex Ultimate 3000 system with a TSQ Quantum Access Max mass spectrometer (ThermoFisher Scientific). Separation was achieved on a Syncronis C18 column (100 × 2.1 mm, 1.7 μm) at 40°C. Chromatographic and MS parameters followed Radović et al. (Radović et al. 2020), with Xcalibur software (v2.2) for control and analysis. Phenolics were identified using commercial standards and quantified as μg/g. The UHPLC–MS/MS analysis included comparisons with standard phenolic compounds (gallic acid, caffeic acid, quercetin, rutin, and apigenin) for calibration, identification, and quantification of polyphenolic compounds in the extracts.

### 2.5 Antioxidant Assays

The antioxidant potential of each extract of ZG was assessed using various methods, including reducing power (FRAP (Benzie and Strain 1996) and CUPRAC (Apak et al. 2004)), radical scavenging (DPPH (Kirby and Schmidt 1997) and ABTS (Re et al. 1999)), and the phosphomolybdenum assay (Prieto et al. 1999). The findings were reported in terms of trolox equivalents (mg TE/g extract). Additionally, the metal chelating power assay (Dinis et al. 1994), another method used to evaluate antioxidant activity, presented its results as milligrams of EDTA equivalent per gram of extract (mg EDTAE/g extract). In the FRAP assay, 20 μL of each extract was added to 180 μL of FRAP reagent, prepared by mixing 300 mM acetate buffer, 10 mM TPTZ in 40 mM HCl, and 20 mM FeCl₃·6H₂O in a 10:1:1 ratio. The mixture was incubated at 37°C for 30 min, and absorbance was recorded at 593 nm. For the CUPRAC assay, 50 μL of the extract was combined with 50 μL each of 10 mM CuCl₂, 7.5 mM neocuproine, and 60 μL ammonium acetate buffer (pH 7.0), followed by 30 min of incubation at room temperature and absorbance measurement at 450 nm. In the DPPH and ABTS radical scavenging assays, extracts were reacted with 0.1 mM DPPH (in methanol) or 7 mM ABTS^+^ (in PBS) for 30 min in the dark, and absorbance was recorded at 517 and 734 nm, respectively. The phosphomolybdenum assay was conducted by mixing the extracts with a reagent containing 0.6 M H₂SO₄, 28 mM Na₃PO₄, and 4 mM (NH₄)₂MoO₄, followed by heating at 95°C for 90 min and absorbance measurement at 695 nm. Trolox (0–1000 μM) was used to construct calibration curves, and all assays were performed with appropriate blanks.

### 2.6 Enzyme Inhibition Studies

The inhibitory potential of each extract against the enzymes acetylcholinesterase (AChE), butyrylcholinesterase (BChE) (Ellman et al. 1961), tyrosinase (Masuda et al. 2005), α‐amylase (Šafašík 1990), and α‐glucosidase (Ting et al. 2005) was evaluated using established in vitro methods. To evaluate AChE and BChE inhibition, the extracts were first incubated with the enzyme (0.2 U/mL) and 0.3 mM DTNB in Tris–HCl buffer (pH 8.0). The reaction was initiated by adding 1.5 mM acetylthiocholine or butyrylthiocholine, and the absorbance was monitored at 412 nm for 10 min. Tyrosinase inhibition was evaluated using 2 mM L‐DOPA in phosphate buffer (pH 6.8), with absorbance measured at 475 nm. For α‐amylase and α‐glucosidase assays, 1% starch and 5 mM p‐nitrophenyl‐α‐D‐glucopyranoside (pNPG) were used as substrates, respectively. Reactions were terminated with sodium carbonate (Na₂CO₃), and absorbance was measured at 540 nm for α‐amylase and 405 nm for α‐glucosidase. Standard inhibitors included galantamine for AChE and BChE, kojic acid for tyrosinase, and acarbose for both α‐amylase and α‐glucosidase.

### 2.7 Evaluation of the Antimicrobial Activity

The antibacterial and antifungal activities of the extracts were assessed using the microdilution method, as outlined in earlier research (Daouk et al. 1995; Lambert et al. 2001). The microbial strains were sourced from the Department of Plant Physiology at the Institute for Biological Research “Siniša Stanković,” National Institute of the Republic of Serbia, University of Belgrade. The antimicrobial activity was evaluated using the microdilution method in accordance with CLSI standards. Extracts were serially diluted (ranging from 1.95 to 1000 μg/mL) in Mueller‐Hinton broth for bacterial strains and RPMI‐1640 medium for fungal strains. Microbial suspensions (1 × 10^5^ CFU/mL) were added, and plates were incubated for 24 h at 37°C for bacteria and 48 h at 30°C for fungi. Growth inhibition was determined using resazurin dye. Ciprofloxacin and fluconazole served as positive controls for bacteria and fungi, respectively. The minimum inhibitory concentration (MIC) was defined as the lowest extract concentration at which no visible microbial growth was observed.

### 2.8 Evaluation of Cytotoxicity on HaCaT Cells

The cytotoxic effects of the extracts on HaCaT cells were evaluated using a crystal violet assay (Ordaz‐Hernández et al. 2024). Cytotoxicity was categorized as: IC50 ≤ 20 μg/mL (highly cytotoxic), 21–200 μg/mL (moderately cytotoxic), 201–400 μg/mL (weakly cytotoxic), and > 401 μg/mL (non‐cytotoxic). HaCaT cells were seeded at a density of 1 × 10^4^ cells per well in 96‐well plates and exposed to various concentrations of the extracts (1–500 μg/mL) for 24 h. After treatment, cells were fixed using methanol and stained with 0.5% crystal violet for 10 min. The bound dye was then solubilized with 1% SDS, and absorbance was recorded at 570 nm.

### 2.9 Virtual Screen

#### 2.9.1 Protein and Ligand Preparation

Molecular docking analyses were conducted to elucidate the interactions between major phytochemicals identified in the extract of *Ziziphora galinae*, namely rutin, hispidulin, 5‐caffeoylquinic acid, apigenin, p‐coumaric acid, quercetin‐3‐O‐glucoside, 3‐O‐caffeoylquinic acid, caffeic acid, and luteolin. The three‐dimensional (3D) structures of the target proteins were retrieved from the Protein Data Bank (PDB, https://www.rcsb.org/) using their respective PDB IDs, based on protein annotations obtained from the UniProt database (https://www.uniprot.org/). AChE (PDB ID: 7E 3H) (Dileep et al. 2022), BChE (PDB ID: 6EQP) (Rosenberry et al. 2017), α‐amylase (PDB ID: 2QV4), tyrosinase (PDB ID: 2QV4) was retrieved instead. Similarly, a homology model of the human glucosidase enzyme (PDB ID: 7KBJ). *E. cloacae* : OmpF (PDB ID: 6ENE) (Acosta‐Gutiérrez et al. 2018), AmpC (PDB ID: 6LC7) (Kawai et al. 2020), D‐alanyl‐D‐alanine Endopeptidase (PDB ID: 6AZI), LptC (PDB ID: 6MIT), LptF (PDB ID: 6MIT), LptG (PDB ID: 6MIT) (Owens et al. 2019), MurA (PDB ID: 3LTH) (Han et al. 2010), Nitroreductase (PDB ID: 1KQB) (Haynes et al. 2002), NMC‐A (PDB ID: 1BUE) (Swarén et al. 1998), OXA‐436 (PDB ID: 7ODA) (Lund et al. 2021). *S. typhimurium* : Gyrase A (C‐Terminal) (PDB ID: 5ZTJ) (Sachdeva et al. 2020), Gyrase A (N‐Terminal) (PDB ID: 8J9T), Gyrase B (PDB ID: 5ZXM) (Gupta et al. 2021), OmpC (PDB ID: 3UU2), RamR (PDB ID: 6IE9) (Yamasaki et al. 2019) and N‐terminal acetyltransferase (PDB ID: 6AZI). *S. aureus* : DHPS (PDB ID: 1 ad4) (Hampele et al. 1997), ParE similar to Gyrase B (PDB ID: 4URN) (Lu et al. 2014), PBP4 (PDB ID: 5TW8) (Alexander et al. 2018), RpsC (PDB ID: 5TCU) (Belousoff et al. 2017), MurE (PDB ID: 4C13) (Wang et al. 2021). *L. monocytogenes* : InlA (PDB ID: 1O6T) (Schubert et al. 2002), PI‐PLC (PDB ID: 1AOD) (Moser et al. 1997), PrfA (PDB ID: 6EXL) (Kulen et al. 2018), InlB (PDB ID: 4J4L), InlC (PDB ID: 1XEU) (Ooi et al. 2006), InlJ (PDB ID: 3BZ5) (Bublitz et al. 2008), LLO (PDB ID: 4CDB) (Köster et al. 2014), InlH (PDB ID: 1H62) (Barna et al. 2001), MurA (PDB ID: 3R38), IspD (PDB ID: 3F1C). All protein structures were prepared using Discovery Studio software.

#### 2.9.2 Docking Grid and Parameters

The docking grid files were generated based on literature data or predicted using the POCASA v1.1 server (https://g6altair.sci.hokudai.ac.jp/g6/service/pocasa/). Grid box centers and dimensions were defined as follows: Human targets: AChE (−54.44, Y: 32.9, −28.65), BChE (32.16, −16.33, 40.73), tyrosinase (21.81, 12.22, 91.40), glucosidase (−18.79, 2.18, 15.75), amylase (14.188, 48.964, 22.886; 28 × 28 × 24 Å) The grid box dimensions were set to 25 × 25 × 25 Å for all targets except amylase.

*Enterobacter cloacae* : OmpF (21.617, 23.814, −19.447; 90 × 100 × 74 Å), AmpC (6.074, 3.304, 14.955; 126 × 60 × 112 Å), D‐alanyl‐D‐alanine Endopeptidase (39.655, 2.545, 12.088; 70 × 70 × 88 Å), LptC (23.603, −119.812, −25.126; 90 × 90 × 90 Å), LptF (−45.705, −39.837, 60.968; 90 × 90 × 90 Å), LptG (−20.137, −21.178, 77.792; 116 × 76 × 60 Å), MurA (51.411, 69.292, 70.201; 64 × 126 × 96 Å), Nitroreductase (12.368, 5.416, 52.333; 104 × 68 × 96 Å), NMC‐A (24.286, 13.575, 14.006; 100 × 90 × 126 Å), OXA‐436 (−27.156, −3.198, 3.918; 86 × 96 × 74 Å). *Salmonella Typhimurium* : RamR (−3.453, −1.338, 10.02; 74 × 112 × 72 Å), N‐terminal acetyltransferase (−30.65, 47.496, 15.765; 56 × 88 × 70 Å), N‐terminal Gyrase A Pose‐1 (12.798, 69.609, −28.236; 40 × 40 × 40 Å), N‐terminal Gyrase A Pose‐2 (12.798, 69.609, −9.427; 40 × 40 × 40 Å), C‐terminal Gyrase A Pose‐1 (24.448, 23.03, 24.901; 46 × 44 × 48 Å), C‐terminal Gyrase A Pose‐2 (27.977, 37.171, 35.761; 30 × 34 × 32 Å), Gyrase B (−9.464, −8.996, 28.863; 94 × 50 × 78 Å). *Staphylococcus aureus* : DHPS (32.46, 6.683, 42.972; 60 × 60 × 60 Å), Gyrase B (31.684, 5.252, 1.572; 60 × 60 × 40 Å), PBP4 (21.390, −62.210, 39.196; 60 × 60 × 60 Å), RpsC (99.46, 230.082, 201.387; 76 × 76 × 76 Å), MurE (−23.122, 2.508, 9.873; 60 × 60 × 60 Å). *Listeria monocytogenes* : PI‐PLC (30.236, 37.512, 21.541; 64 × 94 × 108 Å), PrfA (−17.276, −14.527, 9.533; 40 × 40 × 40 Å), InlA (−7.839, 15.509, 64.698; 88 × 70 × 50 Å), InlB (−11.043, 4.967, −23.943; 62 × 46 × 78 Å), InlC (23.703, 23.065, 15.047; 50 × 40 × 26 Å), InlJ (79.807, −12.073, −97.841; 40 × 40 × 40 Å), LLO (−6.104, 22.138, −52.133; 100 × 76 × 100 Å), InlH (2.075, 9.185, 37.067; 76 × 76 × 76 Å), MurA (18.169, 38.408, 28.905; 54 × 88 × 66 Å), IspD (−16.63, 18.069, −39.052; 90 × 40 × 94 Å). The 3D structures of all ligands were obtained from the PubChem database (https://pubchem.ncbi.nlm.nih.gov/) and geometry‐optimized using Avogadro v1.2.0. Protein and ligand preparations for docking were performed using AutoDockTools in AutoDock v4.2.6 (Trott and Olson 2010).

#### 2.9.3 Validation and Interaction Analysis

Molecular docking studies were performed using AutoDock Vina v1.1.2 (https://autodock.scripts.edu), with the exhaustiveness parameter set to 32 to ensure thorough sampling of ligand conformations. Grid box dimensions were precisely defined based on the coordinates of the identified active sites. To validate the docking protocol, native co‐crystallized ligands were re‐docked into their respective binding pockets, and root‐mean‐square deviation (RMSD) values were calculated to assess prediction accuracy. Protein–ligand interaction profiles were evaluated using the Protein–Ligand Interaction Profiler (PLIP) (https://plip‐tool.biotec.tu‐dresden.de/plip‐web/plip/index), with a particular focus on hydrogen bonding patterns. Molecular interactions were visualized using PyMOL v2.5.8 and ChimeraX v1.7.1 to enhance the interpretation and presentation of docking results.

### 2.10 Molecular Dynamics Simulations

We performed MD simulations using the CHARMM‐GUI interface (https://charmm‐gui.org), following the standard workflow of the Solution Builder module (Kong et al. 2024). Protein–ligand systems were parameterized with the CHARMM36m force field (Maier et al. 2015), solvated in a TIP3P water box, and neutralized with counterions. A 0.15 M NaCl concentration was maintained to mimic physiological conditions. The Verlet cutoff scheme was used for non‐bonded interactions, and the LINCS algorithm was applied for constraining hydrogen‐involving bonds. Long‐range electrostatics were handled via the Particle Mesh Ewald (PME) method. After energy minimization with the steepest descent algorithm (until reaching < 1000 kJ/mol/nm), equilibration was carried out under NVT and NPT ensembles at 310 K. Production simulations (100 ns) were run with GROMACS 2023.3. MD was conducted for complexes of the *E. cloacae* AmpC_Rutin, *E. cloacae* MurA_Rutin, *S. aureus* MurE_Luteolin, *L. monocytogenes* MurA1_Rutin, *S. aureus* MurE_Apigenin, *S. typhimurium Gyrase* B_3‐O‐Caffeoylquinic acid, *S. aureus* PBP4_Rutin, *S. aureus* MurE_Hispidulin, *E. cloacae* MurA_Quercetin_3‐O‐glucoside, *S. typhimurium* Gyrase A_Rutin, *L. monocytogenes* InlA_3‐O‐Caffeoylquinic acid.

### 2.11 Free Energy Calculation

Binding free energy calculations were carried out using the Molecular Mechanics/Poisson‐Boltzmann Surface Area (MM/PBSA) method implemented in the gmx_MMPBSA tool. MM/PBSA free energy calculations were performed with gmx_MM/PBSA (Miller III et al. 2012; Valdés‐Tresanco et al. 2021) on the 100 ns MD trajectories of each complex. This approach enabled the estimation of ligand binding affinities based on van der Waals, electrostatic, polar solvation, and non‐polar solvation energy components.

## 3 Results and Discussion

### 3.1 TPC and TFC

Table 1 shows the TPC and TFC of ZG in two different extracts: infusion and 70% ethanol. The TPC of ZG in the infusion extract is 75.73 mg GAE/g, while the TFC is 8.87 mg RE/g. On the other hand, the TPC of ZG in the 70% ethanolic extract is lower at 10.44 mg GAE/g, and the TFC is also lower at 2.44 mg RE/g. TPC and TFC are commonly used as indicators of the antioxidant activity of plant extracts since phenolic compounds and flavonoids are major contributors to their antioxidant potentials (Dudonne et al. 2009). Higher TPC and TFC levels therefore typically indicate higher antioxidant activity (Aryal et al. 2019; Brighente et al. 2007). Therefore, based on the data in Table 1, the infusion extract of ZG may have a higher antioxidant activity than the 70% ethanolic extract. However, other factors such as the specific types and concentrations of phenolic and flavonoid compounds present in the extracts can also affect the antioxidant activity of the plant extract (Mutha et al. 2021; Roy et al. 2022). The TPC and TFC of ZG in the infusion extract is significantly higher than those of the 70% ethanolic extract, which aligns with previous findings showing that hot water‐based extraction methods can be more efficient at extracting polar phenolics and flavonoids responsible for antioxidant activity (Agafonova et al. 2019; Bhebhe et al. 2016).

**TABLE 1:** Total phenolic and flavonoid contents in the tested extracts.

Extracts	TPC (mg GAE/g)	TFC (mg RE/g)
Infusion	75.73 ± 0.22	8.87 ± 0.36
70% Ethanol	10.44 ± 0.10	2.44 ± 0.17

*Note:* Values are reported as mean ± SD of three parallel measurements. Abbreviations: GAE, Gallic acid equivalent; RE, Rutin equivalent.

### 3.2 UHPLC–MS/MS Quantitative Profiling

Table 2 shows the concentration of various compounds in two different extracts of an ZG. The analysis of the infusion and 70% ethanolic extracts of ZG reveals distinct patterns in the concentration of phenolic acids, flavonoids, and other bioactive compounds. Notably, the 70% ethanolic extract generally shows higher concentrations of key compounds compared to the aqueous extract, particularly in 3‐O‐caffeoylquinic acid (59.85 vs. 19.17 mg/kg), 5‐O‐caffeoylquinic acid (153.24 vs. 43.25 mg/kg), and p‐coumaric acid (1753.87 vs. 552.54 mg/kg). This suggests that 70% ethanol is a more effective solvent for extracting these compounds, making it possible for it to dissolve a variety of bioactive substances found in plant materials (Cordeiro et al. 2022). Nevertheless, the distinct plant matrix and the chemical makeup of the target chemicals can affect how well ethanol works as an extraction solvent (Galanakis et al. 2013). For example, certain compounds like caffeic acid (15.11 mg/kg in aqueous, 8.57 mg/kg in 70% ethanol) and quercetin‐3‐O‐glucoside (52.88 mg/kg in aqueous, 28.50 mg/kg in 70% ethanol) were found in higher concentrations in the infusion extract. Flavonoids like rutin, hispidulin, and vitexin exhibited variable solubility, with rutin showing significantly higher levels in the 70% ethanolic extract (18.33 mg/kg) compared to the aqueous (5.21 mg/kg). These variations highlight the importance of solvent choice in the extraction process because the yield and chemical makeup of bioactive chemicals recovered from plant materials are directly impacted by the polarity, solubility, and selectivity of various solvents (Tella and Oseni 2019). In the studies, the numerous *Ziziphora* plants contain a variety of flavonoids (Sarikurkcu et al. 2019; Šmejkal et al. 2016). *Z. clinopodioides* has been found to contain luteolin, apigenin, chrysin, acacetin, and diosmetin (Zhang et al. 2015). Extracts from *Z. clinopodioides* were discovered to include isoquercetin, rutin, apigenin, hesperidin, and naringenin (Özkan et al. 2020).

**TABLE 2:** Concentrations (μg/g dry weight) of bioactive compounds in ZG infusion and 70% ethanolic extracts, analyzed in triplicate.

Compounds	Infusion	70% Ethanol
3‐O‐Caffeoylquinic_acid	19.17 ± 0.57	59.85 ± 0.57
5‐O‐Caffeoylquinic_acid	43.25 ± 2.78	153.24 ± 10.85
Caffeic_acid	15.11 ± 0.53	8.57 ± 0.54
Isoorientin	3.14 ± 0.25	2.49 ± 0.06
Rutin	5.21 ± 0.10	18.33 ± 0.02
Vitexin	2.42 ± 0.03	1.53 ± 0.07
p‐Coumaric_acid	552.54 ± 10.24	1753.87 ± 22.97
Quercetin_3‐O‐glucoside	52.88 ± 2.39	28.50 ± 1.13
Quercetin_3‐O‐rhamnoside	0.44 ± 0.05	0.60 ± 0.02
Eriodictyol	4.25 ± 0.15	1.57 ± 0.04
Luteolin	10.78 ± 0.23	8.16 ± 0.45
Quercetin	6.53 ± 0.46	2.40 ± 0.15
Naringenin	1.78 ± 0.15	NF
Apigenin	13.10 ± 0.34	6.16 ± 0.27
Kaempferol	6.02 ± 0.13	NF
Hispidulin	5.47 ± 0.06	11.44 ± 0.15
Isorhamnetin	4.70 ± 0.40	3.36 ± 0.17
Chrysin	4.91 ± 0.01	4.22 ± 0.17
Pinocembrin	3.23 ± 0.00	1.96 ± 0.07

### 3.3 Antioxidant Properties

Table 3 shows the antioxidant activities of ZG in two different extracts, infusion, and 70% ethanol. The different methods used to measure antioxidant activity are PBD, DPPH, ABTS, CUPRAC, FRAP, and MCA. The PBD values of the infusion extract of ZG are higher than that of the 70% ethanolic extract. The PBD value of the infusion extract (1.19 mmol TE/g) is more than twice as high as that of the 70% ethanolic extract (0.53 mmol TE/g). Higher PBD values, which are suggestive of superior biological or pharmacological activity, may result from the tendency of infusion extracts, which are prepared with water, to extract more polar molecules like certain flavonoids and polyphenols. However, a wider variety of phytochemicals, including less polar molecules, are frequently extracted by 70% ethanolic extracts, which may reduce the quantity of particular PBD‐related active ingredients (Abubakar and Haque 2020). The DPPH value of the infusion extract (112.11 mg TE/g) is almost 9 times higher than that of the 70% ethanolic extract (12.97 mg TE/g). The ABTS values of the infusion extract of ZG are significantly higher than that of the 70% ethanolic extract. The CUPRAC value of the infusion extract (305.14 mg TE/g) is around 6 times higher than that of the 70% ethanolic extract (51.55 mg TE/g). The FRAP values of the infusion extract of ZG are higher than that of the 70% ethanol extract. The MCA value of the infusion extract (15.08 mg EDTAE/g) is more than 8 times higher than that of the 70% ethanolic extract (1.86 mg EDTAE/g). To summarize, the results suggest that the infusion extract of ZG has a significantly higher antioxidant activity than the 70% ethanolic extract. This is evident in all the different methods used to measure antioxidant activity. The higher antioxidant activity of the infusion extract is likely due to its higher content of phenolic and flavonoid compounds, as indicated by the TPC and TFC values in the previous table 1. According to earlier studies (Sharopov and Setzer 2011; Zhaparkulova et al. 2022), the TPC of ZG infusion (75.73 mg GAE/g) is higher than that of *Z. clinopodioides* (68.2 mg GAE/g), indicating a higher phenolic content and stronger antioxidant activity (Table 3). Numerous studies have assessed the antioxidant abilities of *Ziziphora* species, with particular attention paid to metal chelating activity (MCA), CUPRAC reducing power, FRAP reducing power, DPPH radical scavenging activity, and ABTS radical scavenging activity (Taheri et al. 2023; Tomczyk et al. 2019; Zhaparkulova et al. 2022). The inhibitory effect of the essential oil of *Z. clinopodioides* leaves varied with concentration. In the DPPH, ABTS, FRAP, and MCA tests, methanol and water extracts often show more antioxidant activity than ethyl acetate extracts (Taheri et al. 2023). Water extract exhibits the strongest metal chelating activity, although ethyl acetate and methanol extracts have comparable CUPRAC ability (Tomczyk et al. 2019). The presence of phenolic compounds and components of essential oils in *Ziziphora* species is responsible for these antioxidant properties (Salehi et al. 2005; Shahbazi 2017).

**TABLE 3:** Antioxidant properties of ZG infusion and 70% ethanolic extracts.

Extracts	PBD (mmol TE/g)	DPPH (mg TE/g)	ABTS (mg TE/g)	CUPRAC (mg TE/g)	FRAP (mg TE/g)	MCA (mg EDTAE/g)
Infusion	1.19 ± 0.023	112.11 ± 2.45	238.19 ± 2.55	305.14 ± 3.74	168.34 ± 3.31	15.08 ± 0.37
70% Ethanol	0.53 ± 0.031	12.97 ± 0.87	30.66 ± 0.53	51.55 ± 0.74	29.41 ± 0.42	1.86 ± 0.15

*Note:* Values are reported as mean ± SD of three parallel measurements. Abbreviations: EDTAE, EDTA equivalents; TE, Trolox equivalents.

### 3.4 Enzyme Inhibitory

Table 4 shows the inhibitory effects of ZG on various enzymes, including AChE, BChE, α‐amylase, α‐glucosidase, and tyrosinase. The inhibitory effects were measured for two different extracts of ZG, infusion, and 70% ethanol. The AChE inhibitory activity of the 70% ethanolic extract (2.99 mg GALAE/g) of ZG is almost four times higher than that of the infusion extract (0.82 mg GALAE/g). This suggests that the 70% ethanolic extract may have a higher potential for treating Alzheimer's disease, which is characterized by the loss of cholinergic neurons due to AChE activity. The BChE inhibitory activity of the 70% ethanolic extract (3.16 mg GALAE/g) of ZG is significantly higher than that of the infusion extract, for which no value is reported. BChE is another important enzyme involved in the pathogenesis of Alzheimer's disease, and its inhibition can help prevent the breakdown of neurotransmitters (Greig et al. 2002). The α‐amylase inhibitory activity of the 70% ethanolic extract (0.27 mmol ACAE/g) of ZG is higher than that of the infusion extract (0.06 mmol ACAE/g). α‐amylase is an enzyme involved in the breakdown of carbohydrates, and its inhibition can help prevent hyperglycemia. The α‐glucosidase inhibitory activity of the infusion extract (0.92 mmol ACAE/g) of ZG is slightly higher than that of the 70% ethanolic extract (1.01 mmol ACAE/g). α‐glucosidase is another enzyme involved in the breakdown of carbohydrates, and its inhibition can also help prevent hyperglycemia. The tyrosinase inhibitory activity of the 70% ethanolic extract (74.89 mg KAE/g) of ZG is significantly higher than that of the infusion extract (12.20 mg KAE/g). Tyrosinase is an enzyme involved in the synthesis of melanin, and its inhibition can help prevent hyperpigmentation. These inhibitory characteristics point to ZG extracts' tangible medicinal potential. The dual inhibition of AChE (2.99 mg GALAE/g) and BChE (3.16 mg GALAE/g) by the ethanol extract is similar to the mechanism of FDA‐approved Alzheimer's medications such as rivastigmine, suggesting potential use in the treatment of neurodegenerative diseases. The extract's ability to block the enzymes that break down carbohydrates (α‐amylase: 0.32 mmol ACAE/g; α‐glucosidase: 1.01 mmol ACAE/g) is similar to how well diabetic drugs like acarbose work, which supports its use in glycemic management. Additionally, the impressive tyrosinase inhibition (74.89 mg KAE/g) emphasizes dermatological applications for disorders like melasma and is equivalent to clinical depigmenting treatments like kojic acid. These results provide mechanistic support for the pharmacological development of *Ziziphora* species and are consistent with their traditional applications. Moreover, the results suggest that the 70% ethanolic extract of ZG has a higher inhibitory activity on AChE, BChE, α‐amylase, and tyrosinase enzymes, while the infusion extract has a slightly higher inhibitory activity on α‐glucosidase enzyme (Zolghadri et al. 2019). Due to its higher concentration of flavonoids such as rutin and luteolin, ZG ethanol extract (74.89 mg KAE/g) exhibits better tyrosinase inhibition than *Z. taurica* extracts (70.3 mg KAE/g) (Sarikurkcu et al. 2019). The higher inhibitory activity of the 70% ethanolic extract could be due to the presence of different active compounds, which are better extracted in 70% ethanol than in water. *Ziziphora* species have shown enzyme inhibitory action, particularly against α‐amylase and tyrosinase (Sarikurkcu et al. 2019; Tomczyk et al. 2019). The maximum α‐amylase inhibition is seen in water extracts of *Z. taurica* subsp. *cleonioides*, whereas the lowest is seen in ethyl acetate extracts (Sarikurkcu et al. 2019; Zhaparkulova et al. 2022). Certain flavonoids and other phytochemicals present in *Ziziphora* extracts have been shown to be potent inhibitors of mushroom tyrosinase (Taheri et al. 2023; Zhaparkulova et al. 2022). One important mechanism of action for triterpenes, which are found in *Ziziphora* species, is the inhibition of α‐glucosidase and α‐amylase. Because of its enzyme‐inhibiting qualities (Malaník et al. 2025), *Ziziphora* has long been used to treat ailments like skin diseases and hyperglycemia (Bahadori et al. 2019). The investigation of enzyme inhibition in *Ziziphora* sheds light on possible medicinal uses (Bahadori et al. 2019).

**TABLE 4:** Inhibitory effects of ZG infusion and 70% ethanolic extracts on enzyme activity.

Extracts	AChE (mg GALAE/g)	BChE (mg GALAE/g)	α‐Amylase (mmol ACAE/g)	α‐Glucosidase (mmol ACAE/g)	Tyrosinase (mg KAE/g)
Infusion	0.82 ± 0.078	na	0.06 ± 0.01	0.92 ± 0.01	12.20 ± 0.59
70% Ethanol	2.99 ± 0.018	3.16 ± 0.06	0.27 ± 0.01	1.01 ± 0.01	74.89 ± 0.63

*Note:* Values are reported as mean ± SD of three parallel measurements. Abbreviations: ACAE, Acarbose equivalent; GALAE, Galantamine equivalent; KAE, Kojic acid equivalent.

### 3.5 Antibacterial Activity

This Table 5 displays the findings of a study that looked at how different bacterial strains responded to different treatments in terms of their MIC and minimum bactericidal concentration (MBC). In contrast to MBC values, which show the lowest concentration of the therapy that kills the bacteria, MIC values show the lowest concentration of the treatment that prevents bacterial growth (Kowalska‐Krochmal and Dudek‐Wicher 2021). Table 5 shows the different extracts have varying effectiveness against different bacterial strains. The MIC values for infusions ranged from 0.125 to 3.0 mg/mL, with *E. cloacae* and *S. typhimurium* showing the highest sensitivity (MIC of 0.125 mg/mL), while *M. luteus* exhibited the least susceptibility (MIC of 3.0 mg/mL). The MBC values for the infusions reflected similar trends, with values ranging from 0.25 to 4.0 mg/mL. *S. typhimurium* and *E. cloacae* again had the lowest MBC (0.25 mg/mL), while *M. luteus* required the highest concentration for bactericidal activity (4.0 mg/mL). This suggests that infusions are effective to a certain extent, but higher concentrations are required for complete bacterial elimination, particularly for certain Gram‐positive bacteria like *M. luteus* (Gomes et al. 2001). According to some research, some infusions, like those made with herbs or antimicrobial agents, can stop or lessen the growth of bacteria, both Gram‐positive and Gram‐negative (Pane 2024). 70% ethanolic extracts exhibited stronger antibacterial properties compared to infusions, with lower MIC values across most bacterial strains. MICs for 70% ethanol extracts ranged from 0.25 to 2.0 mg/mL. The lowest MIC (0.25 mg/mL) was recorded for *S. aureus* , while *M. luteus* and *P. aeruginosa* exhibited higher MIC values (2.0 and 1.5 mg/mL, respectively). Similarly, 70% ethanolic extract appears to be more effective against *Listeria monocytogenes* and *Salmonella typhimurium* compared to infusion extract. The MBC values for 70% ethanolic extracts were also lower than those for infusions, with a range from 0.5 to 4.0 mg/mL. For example, *S. aureus* had a MBC of 0.5 mg/mL for 70% ethanolic extract, significantly lower than the infusion's MBC (1.0 mg/mL). According to Table 5, infusion extract is more effective against *Pseudomonas aeruginosa* and less effective against *Bacillus cereus* compared to 70% ethanolic extract, likely due to the higher concentration of active phytochemicals extracted by ethanol. These comparisons highlight the relative effectiveness of natural extracts compared to conventional antibiotics, with ethanol extracts showing more promise than infusions (Palombo 2011). Known for its antibacterial qualities, *Ziziphora* species successfully fight off a variety of infections (Qader et al. 2023). Strong antibacterial action against both Gram‐positive and Gram‐negative bacteria is demonstrated by *Z. clinopodioides* essential oils (Sinaeyan and Sani 2014). By lowering harmful germs and prolonging the shelf life of food items, these essential oils can act as natural preservatives. The methanol extracts and essential oils of *Ziziphora* have broad‐spectrum antibacterial action against microorganisms. Among other therapeutic advantages, the herb has long been utilized for its antibacterial qualities (Greta et al. 2022; Ozturk and Ercisli 2007). According to one of the previous studies, essential oils from *Ziziphora* exhibit antibacterial properties against foodborne pathogens such as *Pseudomonas* sp., *Bacillus* sp., *Escherichia coli* , and *Staphylococcus aureus* (Qader et al. 2023). The plant's essential oils are thought to be responsible for the antibacterial properties (Karimifar et al. 2022). According to (Ozturk and Ercisli 2007), the MIC of ZG ethanol extract against *S. aureus* (0.25 mg/mL) is significantly lower than that of *Z. clinopodioides* (0.5 mg/mL), suggesting increased antibacterial efficacy, perhaps as a result of the synergistic actions of caffeoylquinic acid and rutin (Table 2).

**TABLE 5:** Antibacterial activity of ZG infusion and 70% ethanolic extracts, measured in mg/mL.

Extracts		*S. a*.	*B.c*.	*L. m*.	*M. l*.	*P. ae*.	*E. c*.	*S.t*.	*En. cl*.
Infusion	MIC	0.5	2.0	0.25	3.0	1.5	0.125	0.125	0.5
	MBC	1.0	4.0	0.5	4.0	2.0	0.25	0.25	1.0
70% Ethanol	MIC	0.25	0.5	0.5	2.0	1.5	0.5	0.5	0.5
	MBC	0.5	1.0	1.0	4.0	2.0	1.0	1.0	1.0
Streptomycin	MIC	0.100	0.025	0.150	0.050	0.100	0.100	0.100	0.025
	MBC	0.200	0.050	0.300	0.100	0.200	0.200	0.200	0.050
Ampicillin	MIC	0.100	0.100	0.150	0.100	0.300	0.150	0.100	0.100
	MBC	0.150	0.150	0.500	0.150	0.500	0.200	0.200	0.150

*Note:* Minimum inhibitory concentration (MIC) and minimum bactericidal concentration (MBC) values. Abbreviations: B.c., *Bacillus cereus* ; E.c., *Enterobacter cloacae* ; En.cl, *Enterobacter cloacae* ; M.l., *Micrococcus luteus* ; P.a., *Pseudomonas aeruginosa* ; S.a., *Staphylococcus aureus* ; S.t., *Salmonella typhimurium* .

### 3.6 Antifungal Activity

Table 6 displays the results of a ZG extract analysis that looked at the MFC and MIC of several treatments against distinct fungal strains. With MIC values of 8.0 and 2.0 μg/mL, respectively, ZG infusion extract demonstrated potent inhibitory activities, especially against *Aspergillus fumigatus* and *Aspergillus niger*, demonstrating its possible use in the development of antifungal treatments (Aqil et al. 2010; Sugiarti et al. 2022). With MIC values ranging from 0.375 to 8.0 μg/mL, the extract also demonstrated broad‐spectrum fungistatic and fungicidal activities against other tested fungal strains. The ZG 70% ethanolic extract was efficient against *Penicillium funiculosum* at lower concentrations (MIC = 1.0 μg/mL) and showed increased activity against *A. niger* (MIC = 8.0 μg/mL). The substantial fungicidal potential was further supported by the MFC data, which showed that doses were ≤ 8.0 μg/mL. These results are consistent with earlier research showing that *Ziziphora* species' high phenolic and flavonoid content confers antibacterial effectiveness (Aazza et al. 2011; Salehi et al. 2005). In particular, *Ziziphora* extracts' bioactive flavonoid and phenolic components have been connected to potent antibacterial qualities (Shahbazi 2017). In short, further confirming *Z. galinae*'s antibacterial capabilities are the MIC values found in the current investigation, which are in line with previous studies (Aazza et al. 2011; Salehi et al. 2005; Shahbazi 2017). The findings imply that extracts from ZG, in particular the 70% ethanolic extract, have substantial antifungal action, possibly as a result of the high levels of bioactive phytochemicals they contain. To completely investigate their therapeutic potential, future research should concentrate on the chemical characterization of active ingredients and their mode of action. *T. verrucosum* was inhibited at 1.0 mg/mL by the 70% ethanolic extract, whereas the infusion required 2.0 mg/mL. Additionally, the 70% ethanolic extract achieved fungicidal activity at a lower concentration (2.0 mg/mL) compared to the infusion's 4.0 mg/mL. Similarly, *P. boydii var. catenulata* was more susceptible to both infusions and 70% ethanolic extracts, with ethanol showing a slightly higher MFC (2.0 mg/mL) compared to the infusion (0.5 mg/mL), yet it remained more sensitive overall. Conversely, *A. fumigatus* and *A. niger* exhibited higher resistance to both treatments, requiring higher concentrations for both MIC and MFC, with *A. fumigatus* needing ≥ 8.0 mg/mL for fungicidal activity in both cases. The results underscore the importance of solvent selection in maximizing the extraction of antifungal compounds from natural sources, with 70% ethanol proving to be a more effective medium than water for inhibiting and eradicating fungal strains. Significant antifungal qualities are exhibited by *Ziziphora* species; essential oils from species such as *Z. clinopodioides* and *Z. tenuior* show inhibitory actions against a variety of fungi, including *Aspergillus* and *Candida* species. Pulegone and menthone are two examples of oxygen‐containing monoterpenes that support these antifungal properties (Ćavar Zeljković et al. 2022). *Ziziphora* essential oils have long been used as antiseptics to treat infectious disorders, and clinical research has shown that they are effective against *Candida albicans* (Moghadam et al. 2016).

**TABLE 6:** Antifungal activity of ZG infusion and 70% ethanolic extracts, measured in mg/mL.

		*A. f*.	*A. n*.	*A. v*.	*A. fl*.	*T.v*.	*P.f*.	*P.o*.	*P.v.c*.
Infusion	MIC	8.0	2.0	2.0	2.0	2.0	2.0	2.0	0.375
	MFC	≥ 8.0	4.0	4.0	4.0	4.0	4.0	4.0	0.5
70% Ethanol	MIC	4.0	8.0	2.0	2.0	1.0	2.0	2.0	1.5
	MFC	8.0	≥ 8.0	4.0	4.0	2.0	4.0	4.0	2.0

*Note:* Minimum inhibitory concentration (MIC) and minimum fungicidal concentration (MFC) values. Abbreviations: A.f.: *Aspergillus fumigatus*; A.fl.: *Aspergillus flavus*; A.n.: *Aspergillus niger*; A.v.: *Aspergillus versicolor*; P.f.: *Penicillium funiculosum*; P.o.: *Penicillium ochrochloron*; P.v.c.: *Penicillium verrucosum* var. *cyclopium*; T.v.: *Trichoderma viride*.

Although noteworthy, the antifungal effectiveness of ZG ethanol extracts indicates potential for improvement using different solvent systems. Solvents with different polarities may improve fungal inhibition, according to previous research on related Lamiaceae species. For example, acetone extracts have demonstrated enhanced efficacy against Aspergillus species because they better extract non‐polar terpenoid chemicals (Mohammadhosseini 2017). By obtaining 2–4 times lower MIC values than ethanol extracts in certain medicinal plants, methanol‐chloroform mixtures (1:1 ratio) have shown exceptional efficacy against dermatophytes. This is probably due to the synergistic extraction of both hydrophilic and lipophilic bioactive constituents (Karimifar et al. 2022). By releasing bound phenolic chemicals into their more physiologically active free forms, more sophisticated extraction methods such as subcritical water extraction at regulated temperatures (120°C–180°C) may increase antifungal effectiveness even further (Taheri et al. 2023). Together, these results imply that systematic solvent tuning may be able to preserve the broad‐spectrum activity shown against more susceptible species while circumventing the resistance patterns observed in difficult fungal strains such as *A. fumigatus* . For ZG's possible antifungal applications, future research comparing various extraction techniques would aid in determining the ideal ratio of solvent efficacy, safety, and usefulness.

### 3.7 Cytotoxic Results

HaCaT cell lines were widely utilized for in vitro toxicity assessments using ZG infusion and 70% ethanolic extracts. Evaluating extract‐induced cytotoxicity is a crucial aspect of biomedical research and plays a key role in the bioactive molecule selection process (Canga et al. 2022; Mervin et al. 2016). Moreover, toxicity studies on cultured human cells serve as an essential initial step in developing new possible anticancer agents. In this study, the cytotoxic effects of the extracts of ZG were examined using the HaCaT cell line, an immortalized human keratinocyte model commonly employed for preliminary safety screenings. The results indicated that none of the tested extracts of ZG exhibited cytotoxicity toward this cell line, with IC₅₀ values exceeding 400 μg/mL. However, further investigations on various cancer cell lines are necessary, as the extracts may demonstrate selective cytotoxic effects.

### 3.8 Virtual Screen Results

**FIGURE 1:**
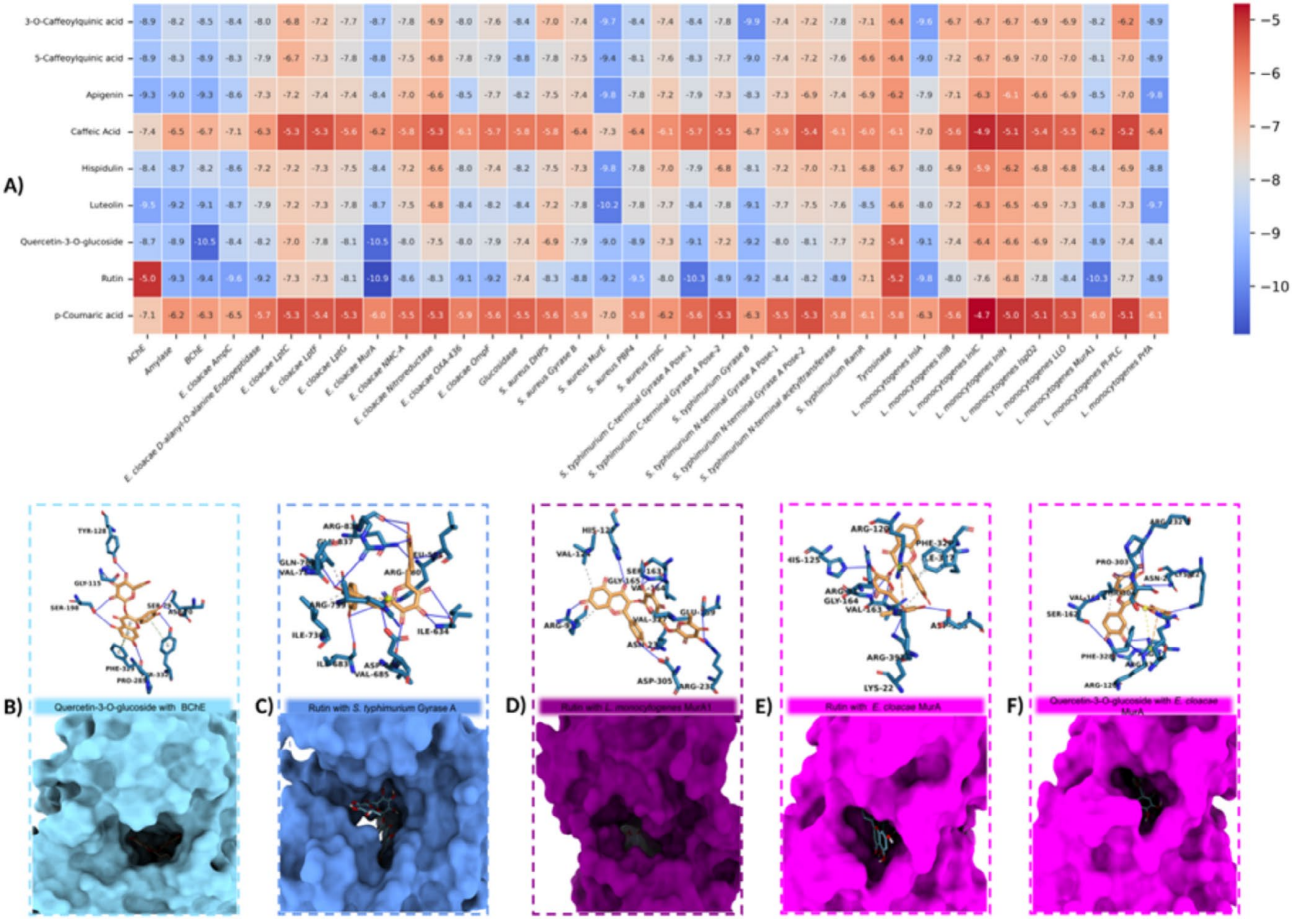
A comprehensive analysis of the binding interactions between proteins and the selected compounds: (A) a graphical representation of docking scores for relevant proteins. (B) Molecular interaction analysis of quercetin‐3‐O‐glucosidewith with BChE. (C) Molecular interaction analysis of rutin with *S. typhimurium* Gyrase A. (D) Molecular interaction analysis of rutin with *L. monocytogenes* MurA1. (E) Molecular interaction analysis of rutin with *E. cloacae* MurA. (F) Molecular interaction analysis of quercetin‐3‐O‐glucoside with *E. cloacae* MurA.

Molecular docking simulations were carried out to evaluate the binding affinities of hub‐like phytochemicals derived from *ZG* against selected bacterial target proteins and standard human enzymes. In this context, natural compounds including rutin, hispidulin, 5‐caffeoylquinic acid, apigenin, *p*‐coumaric acid, quercetin‐3‐O‐glucoside, 3‐O‐caffeoylquinic acid, caffeic acid, and luteolin were tested against protein targets from *Enterobacter cloacae* , *Listeria monocytogenes* , *Salmonella typhimurium* , and *Staphylococcus aureus* , as well as human enzymes such as AChE, BChE, glucosidase, amylase, and tyrosinase. Among the docked complexes, the strongest binding affinities were observed in quercetin‐3‐O‐glucoside‐BChE (−10.5 kcal/mol), rutin‐ *E. cloacae* MurA (−10.9 kcal/mol), rutin‐ *L. monocytogenes* MurA1 (−10.3 kcal/mol), and rutin‐ *S. typhimurium* Gyrase A (−10.3 kcal/mol) (Figure 1B–F). Rutin's potential as a broad‐spectrum bacterial enzyme inhibitor is shown by the fact that its binding energy to *E. cloacae* MurA is (−10.9 kcal/mol). RMSD values obtained from the docking simulations ranged between 0.1 and 7.3 Å, reflecting both conformational stability and flexibility of the ligand‐protein complexes. For further computational evaluation, compound selection was based on three primary criteria: a binding affinity of −9 kcal/mol or lower, an RMSD value ≤ 2 Å, and the formation of at least four Hbonds. The relevant findings are presented in Table 7 and Figure 1A. It should be noted, however, that the exclusion of other compounds from this selection does not imply a lack of inhibitory potential. For instance, meaningful binding affinities were also observed for 3‐O‐caffeoylquinic acid‐AChE (−8.9 kcal/mol), 5‐caffeoylquinic acid‐AChE (−8.9 kcal/mol), quercetin‐3‐O‐glucoside‐amylase (−8.9 kcal/mol), caffeoylquinic acid (−8.9 kcal/mol), 5‐caffeoylquinic acid (−8.4 kcal/mol), and hispidulin‐AChE (−8.3 kcal/mol), respectively. In summary, all compounds with binding energies of −7 kcal/mol or lower can be considered potential inhibitors under suitable biological conditions (Figure 1A).

**TABLE 7:** The docking score (kcal/mol) and interacting residues of the protein.

Compound	Target	PDB ID	Binding energy	RMSD	Interaction		Binding site
					Type	Number	
Rutin	*Amylase*	2QV4	−9.3	0.1	Hbond	11	GLN A:63; ARG A:195; ALA A:198; LYS A:200; GLU A:233; GLU A:233; ILE A:235; HIS A:299; ASP A:300; HIS A:305; HIS A:305
Luteolin	*Amylase*	2QV4	−9.2	1.0	Hbond	6	GLN A:63; ARG A:195; ARG A:195; ASP A:197; GLU A:233; HIS A:299
Apigenin	*AChE*	7E3H	−9.3	1.0	Hbond	3	TRP A: 86; GLY A: 120; TYR A: 133
Luteolin	*AChE*	7E3H	−9.5	0.5	Hbond	6	ASP A:74; ASN A:87; GLY A:120; TYR A:133; GLU A:202; TYR A:337
Rutin	*BChE*	6EQP	−9.4	0.8	Hbond	9	ASP A:70; ASP A:70; TRP A:82; GLY A:115; THR A:120; THR A:120; GLU A:197; TRP A:430; GLY A:439
Apigenin	*BChE*	6EQP	−9.3	0.4	Hbond	2	GLY A:115; GLU A:197
Quercetin‐3‐O‐glucoside	*BChE*	6EQP	−10.5	0.8	Hbond	10	ASP A:70; SER A:79; GLY A:115; GLY A:115; TYR A:128; SER A:198; SER A:198; SER A:198; PRO A:285; TYR A:332
Luteolin	*BChE*	6EQP	−9.1	0.9	Hbond	3	GLY A:115; TYR A:128; LEU A:286
Rutin	*S. typhimurium Gyrase B*	5ZXM	−9.2	6.4	Hbond	8	ASP A:29; ASP A:29; ASP A:29; ASP A:32; THR A:34; HIS A:38; ASP A:338; ASP A:338
5‐Caffeoylquinic acid	*S. typhimurium Gyrase B*	5ZXM	−9	0.6	Hbond	10	ASN A:46; GLU A:50; ASP A:73; GLY A:77; GLY A:77; HIS A:116; GLY A:117; VAL A:118; GLY A:119; THR A:165
Quercetin‐3‐O‐glucoside	*S. typhimurium Gyrase B*	5ZXM	−9.2	0.6	Hbond	7	ASN A:46; GLU A:50; GLY A:114; GLY A:117; VAL A:120; THR A:165; THR A:165
3‐O‐Caffeoylquinic acid	*S. typhimurium Gyrase B*	5ZXM	−9.9	0.1	Hbond	14	ASN A:46; ASP A:73; ARG A:76; GLY A:77; GLY A:77; GLY A:102; GLY A:114; HIS A:116; GLY A:117; VAL A:118; GLY A:119; VAL A:120; SER A:121; THR A:165
Luteolin	*S. typhimurium Gyrase B*	5ZXM	−9.1	0.9	Hbond	6	GLY A:77; GLY A:102; LYS A:103; VAL A:120; THR A:165; THR A:165
Rutin	*S. typhimurium Gyrase A*	5ZTJ	−10.3	0.5	Hbond	15	ARG A:580; LEU A:581; ILE A:634; ILE A:634; ILE A:634; ILE A:683; VAL A:685; ASP A:686; ARG A:739; GLN A:788; GLN A:837; ARG A:838; ARG A:838; ARG A:838; ARG A:838
Quercetin‐3‐O‐glucoside	*S. typhimurium Gyrase A*	5ZTJ	−9.1	1.0	Hbond	15	ASP A:579; ARG A:580; ARG A:580; LEU A:581; LEU A:581; ILE A:634; ASP A:686; ASP A:686; ASP A:686; ILE A:737; ARG A:739; GLN A:788; GLN A:837; GLN A:837; ARG A:838
Rutin	*S. aureus MurE*	4C13	−9.2	6.8	Hbond	14	LYS A:114; ASN A:151; THR A:152; THR A:152; THR A:152; ARG A:187; ARG A:187; HIS A:205; TYR A:351; TYR A:351; ARG A:383; ARG A:383; ASP A:406; ASN A:407
Hispidulin	*S. aureus MurE*	4C13	−9.8	0.9	Hbond	8	THR A:111; GLY A:113; LYS A:114; LYS A:114; THR A:115; THR A:115; SER A:116; ARG A:335
5‐Caffeoylquinic acid	*S. aureus MurE*	4C13	−9.4	1.0	Hbond	7	LYS A:114; THR A:115; THR A:115; SER A:116; HIS A:205; ASN A:301; HIS A:353
Apigenin	*S. aureus MurE*	4C13	−9.8	0.7	Hbond	6	LYS A:114; SER A:116; SER A:116; THR A:137; ASN A:301; ASP A:350
Quercetin‐3‐O‐glucoside	*S. aureus MurE*	4C13	−9	6.6	Hbond	14	LYS A:114; LYS A:114; THR A:115; THR A:115; ASN A:151; GLU A:177; ASP A:204; HIS A:205; TYR A:351; HIS A:353; ARG A:383; ARG A:383; ASP A:406; ASN A:407
3‐O‐Caffeoylquinic acid	*S. aureus MurE*	4C13	−9.7	0.1	Hbond	9	GLY A:113; LYS A:114; THR A:115; THR A:115; THR A:115; THR A:115; SER A:116; ASN A:301; HIS A:353
Luteolin	*S. aureus MurE*	4C13	−10.2	0.1	Hbond	9	LYS A:114; LYS A:114; THR A:115; THR A:115; THR A:115; SER A:116; ASN A:301; ASN A:301; GLY A:357
Rutin	*S. aureus PBP4*	5TW8	−9.5	0.0	Hbond	11	SER A:75; SER A:75; SER A:75; GLU A:114; GLU A:114; ASN A:138; SER A:139; GLU A:183; THR A:260; GLY A:261; SER A:262
Rutin	*L. monocytogenes MurA1*	3R38	−10.3	0.1	Hbond	11	ASN A:23; ARG A:93; HIS A:127; SER A:163; SER A:163; VAL A:164; GLY A:165; GLU A:189; ARG A:233; ARG A:233; ASP A:305
Rutin	*L. monocytogenes InlA*	1O6T	−9.8	7.3	Hbond	15	LYS A:425; LYS A:425; SER A:429; SER A:429; SER A:429; SER A:429; SER A:429; SER A:429; SER A:429; SER A:429; THR A:454; THR A:454; THR A:454; ASP A:457; THR A:459
5‐Caffeoylquinic acid	*L. monocytogenes InlA*	1O6T	−9	0.5	Hbond	14	LYS A:425; LYS A:425; LYS A:425; SER A:429; SER A:429; SER A:429; THR A:454; THR A:454; THR A:454; GLU A:455; PRO A:456; ASP A:457; ASP A:457; ASP A:457
Quercetin 3‐O‐glucoside	*L. monocytogenes InlA*	1O6T	−9.1	0.1	Hbond	13	LYS A:425; SER A:429; SER A:429; SER A:429; SER A:429; SER A:429; SER A:429; THR A:454; THR A:454; ASP A:457; ASP A:457; ASP A:457; THR A:459
3‐O‐Caffeoylquinic acid	*L. monocytogenes InlA*	1O6T	−9.6	0.3	Hbond	15	LYS A:425; LYS A:425; LYS A:425; VAL A:428; SER A:429; SER A:429; SER A:429; THR A:454; THR A:454; GLU A:455; GLU A:455; ASP A:457; ASP A:457; ASP A:457; ASP A:457
Apigenin	*L. monocytogenes PrfA*	1O6T	−9.8	0.7	Hbond	1	TYR A:154
Luteolin	*L. monocytogenes PrfA*	6EXL	−9.7	0.4	Hbond	1	LYS A:122
Rutin	*E. cloacae OXA‐436*	7ODA	−9.1	1.0	Hbond	7	SER A: 70; LYS A: 116; LYS A: 208; THR A: 209; THR A: 209; ARG A: 250; ARG A: 250
Rutin	*E. cloacae MurA*	3LTH	−10.9	0.5	Hbond	8	LYS A:22; ARG A:91; ARG A:91; HIS A:125; VAL A:163; GLY A:164; ASP A:305; ARG A:397
Quercetin‐3‐O‐glucoside	*E. cloacae MurA*	3LTH	−10.5	0.8	Hbond	12	LYS A:22; ASN A:23; ARG A:91; ARG A:91; ARG A:120; ARG A:120; SER A:162; ARG A:232; ARG A:232; THR A:304; ARG A:331; ARG A:331
Rutin	*E. cloacae D‐alanyl‐D‐alanine Endopeptidase*	6AZI	−9.2	1.1	Hbond	15	SER A:68; SER A:68; ARG A:105; ARG A:105; ARG A:105; SER A:125; ASN A:127; ASN A:127; ARG A:215; ARG A:215; ARG A:215; ARG A:215; THR A:217; PHE A:235; PHE A:235
Rutin	*E. cloacae AmpC*	6LC7	−9.6	0.4	Hbond	20	SER A:64; SER A:64; LYS A:67; GLN A:120; TYR A:150; ASN A:152; ARG A:204; ARG A:204; ARG A:204; ARG A:204; ARG A:204; SER A:212; ASN A:289; THR A:314; SER A:316; SER A:316; GLY A:318; GLY A:318; SER A:341; ASN A:344
Rutin	*E. cloacae OmpF*	6ENE	−9.2	6.7	Hbond	12	ASN A:26; ASN A:29; GLY A:114; ASP A:116; ARG A:234; TYR A:283; GLN A:303; GLN A:303; ASN A:305; ASN A:305; ASN A:316; ASN A:316

For AChE, *luteolin* emerged as a notable candidate, establishing 6 Hbonds and exhibiting a binding energy of −9.5 kcal/mol. Against amylase, rutin (11 Hbonds, −9.3 kcal/mol) and *luteolin* (6 Hbonds, −9.2 kcal/mol) displayed strong interactions. Notably, rutin targeted GLN A:63, ARG A:195, ALA A:198, LYS A:200, GLU A:233, ILE A:235, HIS A:299, and ASP A:300, whereas luteolin interacted with ARG A:195, ASP A:197, and GLU A:233. In BChE, *rutin* (9 Hbonds, −9.4 kcal/mol) and *quercetin 3‐O‐glucoside* (10 Hbonds, −10.5 kcal/mol) demonstrated the highest affinities, with the latter's markedly negative binding energy indicating strong inhibitory potential.

Among the *E. cloacae* targets, *rutin* showed broad and robust binding profiles. Specifically, it bound to AmpC with 20 Hbonds (−9.6 kcal/mol) and to the D‐Ala‐D‐Ala endopeptidase with 15 Hbonds (−9.2 kcal/mol). Against OXA‐436, rutin formed 7 Hbonds (−9.1 kcal/mol). Additionally, in MurA, both rutin (8 Hbonds, −10.9 kcal/mol) and *quercetin 3‐O‐glucoside* (12 Hbonds, −10.5 kcal/mol) stood out, targeting residues such as LYS A:22, ARG A:91, and HIS A:125. These results collectively underscore the multi‐site binding capabilities of rutin and quercetin derivatives in diverse *E. cloacae* enzymes.

In *L. monocytogenes* , InlA was strongly bound by *5‐caffeoylquinic acid* (14 Hbonds, −9.0 kcal/mol), *quercetin 3‐O‐glucoside* (13 Hbonds, −9.1 kcal/mol), and *3‐O‐caffeoylquinic acid* (15 Hbonds, −9.6 kcal/mol). The key residues LYS A:425, SER A:429, THR A:454, and ASP A:457 served as common “hot spots” for these three ligands. In MurA1, *rutin* (11 Hbonds, −10.3 kcal/mol) again exhibited high binding affinity. These findings suggest that InlA and MurA1 may harbor conserved binding pockets suitable for caffeoylquinic acid derivatives, as well as rutin‐ and quercetin‐like flavonoids.

For *S. aureus* , multiple flavonoids displayed potent interaction against MurE: *hispidulin* (8 Hbonds, −9.8 kcal/mol), *5‐caffeoylquinic acid* (7 Hbonds, −9.4 kcal/mol), *apigenin* (6 Hbonds, −9.8 kcal/mol), *3‐O‐caffeoylquinic acid* (9 Hbonds, −9.7 kcal/mol), and *luteolin* (9 Hbonds, −10.2 kcal/mol). In particular, luteolin's binding energy of −10.2 kcal/mol was noteworthy. Residues LYS A:114, THR A:115, SER A:116, and ASN A:301 emerged as potential “hot spots” commonly targeted by these ligands. Against PBP4, *rutin* (11 Hbonds, −9.5 kcal/mol) demonstrated considerable affinity, suggesting that *S. aureus* enzymes can be inhibited by a diverse set of phenolic and flavonoid compounds, particularly near the MurE locus, which appears to be a prime region for inhibitor design.

Finally, *S. typhimurium* Gyrase A displayed notable binding with *rutin* (15 Hbonds, −10.3 kcal/mol) and *quercetin 3‐O‐glucoside* (15 Hbonds, −9.1 kcal/mol). Key residues such as ARG A:580, LEU A:581, ILE A:634, and ASP A:686 were involved in multiple interactions. Gyrase B was targeted effectively by *5‐caffeoylquinic acid* (10 Hbonds, −9.0 kcal/mol), *quercetin 3‐O‐glucoside* (7 Hbonds, −9.2 kcal/mol), *3‐O‐caffeoylquinic acid* (14 Hbonds, −9.9 kcal/mol), and *luteolin* (6 Hbonds, −9.1 kcal/mol). Particularly, *3‐O‐caffeoylquinic acid* formed 14 Hbonds (−9.9 kcal/mol) and targeted residues such as ASN A:46, GLY A:77, GLY A:117, THR A:165, and VAL A:120, indicating a likely inhibitor “pocket.”

Taken together, these findings demonstrate that rutin, quercetin 3‐O‐glucoside, 3‐O‐caffeoylquinic acid, luteolin, and related polyphenolic frameworks can form multiple Hbonds in the active sites of diverse enzymes, exhibiting moderate to highly negative binding energies. Ligands with energies at or below −10 kcal/mol, in particular, indicate strong inhibitory prospects. Furthermore, the repeated targeting of the same residues (e.g., LYS A:114, THR A:115, SER A:116) by multiple ligands highlights valuable starting points for pharmacophore modeling and structure‐based drug design. Collectively, our results reveal that these naturally derived phenolic and flavonoid compounds have the potential to serve as broad‐spectrum enzyme inhibitors against both human and bacterial targets, thus warranting further in vitro and in vivo validation to confirm their therapeutic applications.

### 3.9 Molecular Dynamics Simulation Results

In this study, molecular dynamics (MD) simulations were conducted for 100 ns to analyze the complexes formed between seven different bacterial target proteins and natural compounds. The structural and interactional stability of the systems was evaluated using a variety of criteria, including RMSD, root‐mean‐square fluctuation (RMSF), solvent accessible surface area (SASA), minimum distance, and Hbond criteria.

**FIGURE 2:**
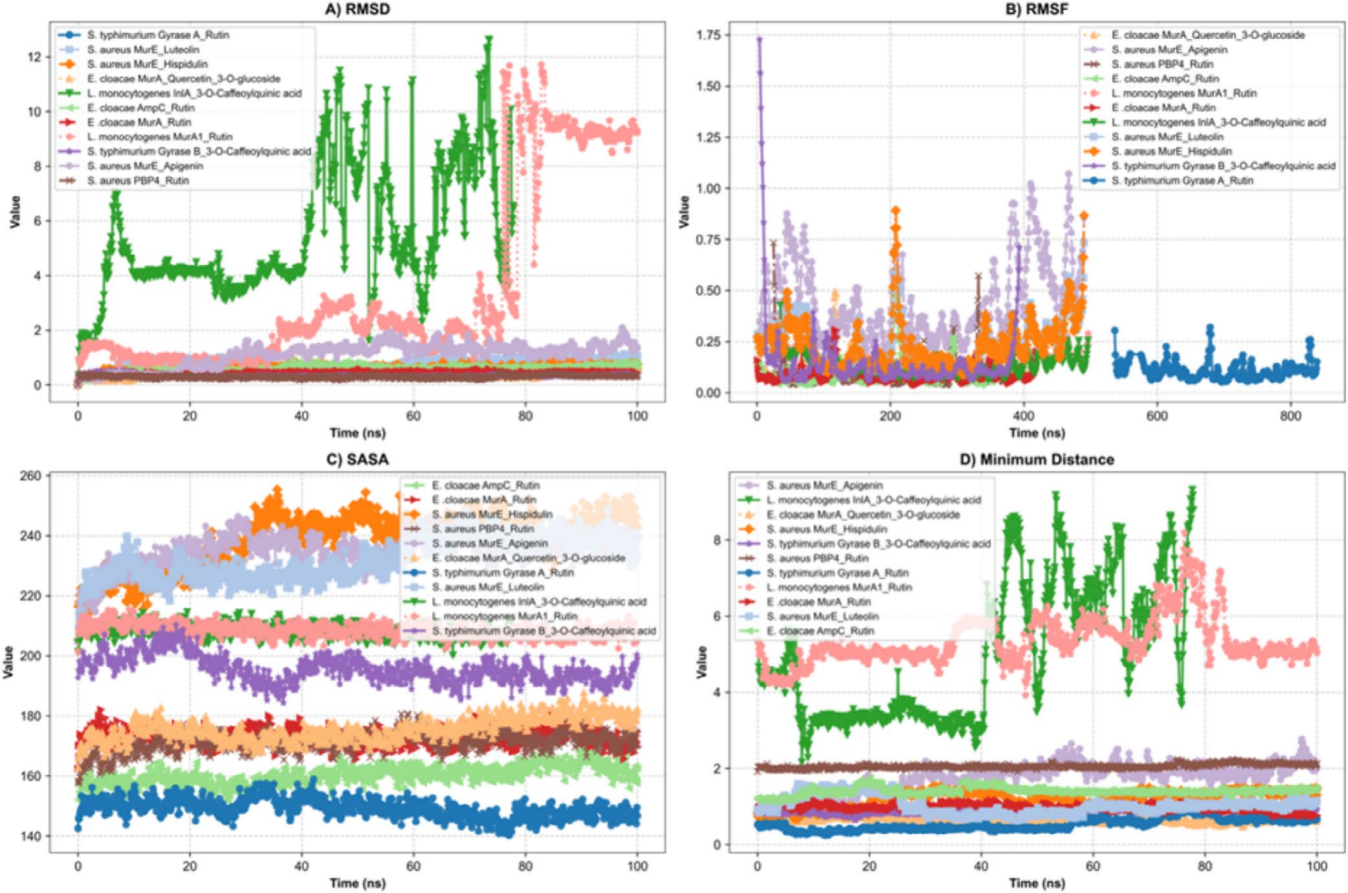
Representation of molecular dynamics simulations in graphical form; (A) RMSD of the *E. cloacae* AmpC_Rutin complex, *E. cloacae* MurA_Rutin complex, *S. aureus* MurE_Luteolin complex, *L. monocytogenes* MurA1_Rutin complex, *S. aureus* MurE_Apigenin complex, *S. typhimurium* Gyrase B_3‐O‐Caffeoylquinic acid complex, *S. aureus* PBP4_Rutin complex, *S. aureus* MurE_Hispidulin complex, *E. cloacae* MurA_Quercetin_3‐O‐glucoside complex, *S. typhimurium* Gyrase A_Rutin complex, and *L. monocytogenes* InlA_3‐O‐Caffeoylquinic acid complex. (B) RMSF of the *E. cloacae* AmpC_Rutin complex, *E. cloacae* MurA_Rutin complex, *S. aureus* MurE_Luteolin complex, *L. monocytogenes* MurA1_Rutin complex, *S. aureus* MurE_Apigenin complex, *S. typhimurium* Gyrase B_3‐O‐Caffeoylquinic acid complex, *S. aureus* PBP4_Rutin complex, *S. aureus* MurE_Hispidulin complex, *E. cloacae* MurA_Quercetin_3‐O‐glucoside complex, *S. typhimurium* Gyrase A_Rutin complex, and *L. monocytogenes* InlA_3‐O‐Caffeoylquinic acid complex. (C) Solvent accessibility of the *E. cloacae* AmpC_Rutin complex, *E. cloacae* MurA_Rutin complex, *S. aureus* MurE_Luteolin complex, *L. monocytogenes* MurA1_Rutin complex, *S. aureus* MurE_Apigenin complex, *S. typhimurium* Gyrase B_3‐O‐Caffeoylquinic acid complex, *S. aureus* PBP4_Rutin complex, *S. aureus* MurE_Hispidulin complex, *E. cloacae* MurA_Quercetin_3‐O‐glucoside complex, *S. typhimurium* Gyrase A_Rutin complex, and *L. monocytogenes* InlA_3‐O‐Caffeoylquinic acid complex. (D) Minimum distance of the *E. cloacae* AmpC_Rutin complex, *E. cloacae* MurA_Rutin complex, *S. aureus* MurE_Luteolin complex, *L. monocytogenes* MurA1_Rutin complex, *S. aureus* MurE_Apigenin complex, *S. typhimurium* Gyrase B_3‐O‐Caffeoylquinic acid complex, *S. aureus* PBP4_Rutin complex, *S. aureus* MurE_Hispidulin complex, *E. cloacae* MurA_Quercetin_3‐O‐glucoside complex, *S. typhimurium* Gyrase A_Rutin complex, and *L. monocytogenes* InlA_3‐O‐Caffeoylquinic acid complex.

The structural stability of the protein–ligand complexes over time was assessed through RMSD analysis. Average RMSD values and their temporal profiles were used to evaluate the extent to which ligands remained confined within their respective binding pockets during the simulations. The *S. typhimurium* Gyrase B_3‐O‐Caffeoylquinic acid complex exhibited remarkable stability, with an average RMSD of 0.34 Å. The *S. typhimurium* Gyrase A_Rutin complex showed a gradual increase in RMSD, reaching an average of 0.63 Å. For the *S. aureus* MurE_Hispidulin complex, a relatively stable trajectory was observed (average 0.61 Å), though an upward trend occurred after 40 ns. The *S. aureus* MurE_Apigenin complex, despite initial low RMSD values, experienced a marked increase to 1.15 Å between 80 and 100 ns. Moderate structural stability was observed in the *S. aureus* MurE_Luteolin complex (average 0.77 Å). Among the most stable systems was the *S. aureus* PBP4_Rutin complex, with a mean RMSD of 0.31 Å. Conversely, significant deviations were recorded for *L. monocytogenes* MurA1_Rutin and *L. monocytogenes* InlA_3‐O‐Caffeoylquinic acid complexes, with average RMSD values of 3.42 Å and 5.42 Å, respectively. The *E. cloacae* MurA_Rutin and MurA_Quercetin_3‐O‐glucoside complexes retained high structural integrity, with respective RMSD values of 0.44 Å and 0.45 Å, while *E. cloacae* AmpC_Rutin showed a slight increase (average 0.59 Å) but remained overall stable. Collectively, most complexes displayed high structural stability (RMSD < 1 Å), with notable exceptions observed in *L. monocytogenes* complexes, where significant structural rearrangements were evident (Figure 2A).

RMSF analysis was employed to assess the local flexibility of protein residues, particularly around ligand binding sites. RMSF values exceeding 0.5 Å typically reflect elevated atomic mobility. In the *S. typhimurium* Gyrase B_3‐O‐Caffeoylquinic acid complex, pronounced flexibility was observed between residues 4 and 8, reaching up to 1.73 Å. In contrast, the *S. typhimurium* Gyrase A_Rutin complex showed high rigidity, with a maximum RMSF of 0.32 Å. The *S. aureus* MurE_Hispidulin complex exhibited moderate fluctuations near the binding pocket (up to 0.89 Å), while the *S. aureus* MurE_Apigenin complex displayed the highest localized flexibility, exceeding 1.00 Å. The *S. aureus* MurE_Luteolin complex reached 0.73 Å in specific regions. The *S. aureus* PBP4_Rutin complex maintained low RMSF values, indicating a rigid structural framework. Both *L. monocytogenes* MurA1_Rutin and InlA_3‐O‐Caffeoylquinic acid complexes demonstrated low flexibility (0.36–0.43 Å). Complexes of *E. cloacae* —namely MurA_Rutin, MurA_Quercetin_3‐O‐glucoside, and AmpC_Rutin—exhibited strong rigidity with average RMSF values between0.08–0.12 Å. Overall, the highest residue‐level fluctuations were recorded in *S. typhimurium* Gyrase B_3‐O‐Caffeoylquinic acid and *S. aureus* MurE_Apigenin, while rigid binding pocket dynamics dominated the remaining complexes, consistent with stable binding profiles (Figure 2B).

SASA analysis revealed differences in solvent exposure before and after ligand binding. The *S. typhimurium* Gyrase B_3‐O‐Caffeoylquinic acid complex maintained a consistent SASA of approximately 195.5 nm^2^ throughout the simulation. A notably low surface area was measured for the *S. typhimurium* Gyrase A_Rutin complex (average 149.2 nm^2^). The *S. aureus* MurE_Hispidulin complex exhibited a maximum SASA of 240.2 nm^2^, suggesting conformational expansion. Similarly, increasing SASA profiles were observed in *S. aureus* MurE_Apigenin and MurE_Luteolin complexes. A moderate increase was recorded in the *S. aureus* PBP4_Rutin complex, stabilizing around 171.6 nm^2^. Minimal surface changes were noted in the *L. monocytogenes* MurA1_Rutin and InlA_3‐O‐Caffeoylquinic acid complexes. Complexes involving *E. cloacae* (MurA_Rutin, MurA_Quercetin_3‐O‐glucoside, and AmpC_Rutin) exhibited stable or slightly increasing SASA values. The observed increases in the *S. aureus* MurE_Hispidulin, MurE_Apigenin, and MurE_Luteolin complexes indicate ligand‐induced conformational flexibility of the protein surface (Figure 1C).

The minimum distance between the ligand and binding site residues was tracked to evaluate spatial retention. Tight binding was maintained throughout the simulations by *S. typhimurium* Gyrase B_3‐O‐Caffeoylquinic acid (0.88 Å), *S. typhimurium* Gyrase A_Rutin (0.53 Å), *E. cloacae* MurA_Rutin (0.98 Å), and MurA_Quercetin_3‐O‐glucoside (0.65 Å), all displaying 100% interaction occupancy. Intermediate distances were noted in *S. aureus* MurE_Hispidulin (1.19 Å), MurE_Apigenin (1.75 Å), and MurE_Luteolin (1.03 Å). A weaker interaction was observed in *S. aureus* PBP4_Rutin (2.06 Å). In contrast, distances exceeding 5 Å were measured for *L. monocytogenes* MurA1_Rutin and InlA_3‐O‐Caffeoylquinic acid, indicating a loss of binding site engagement. These results suggest superior spatial binding stability in *S. typhimurium* and *E. cloacae* complexes, with *L. monocytogenes* systems exhibiting unstable binding behavior (Figure 2D).

**FIGURE 3:**
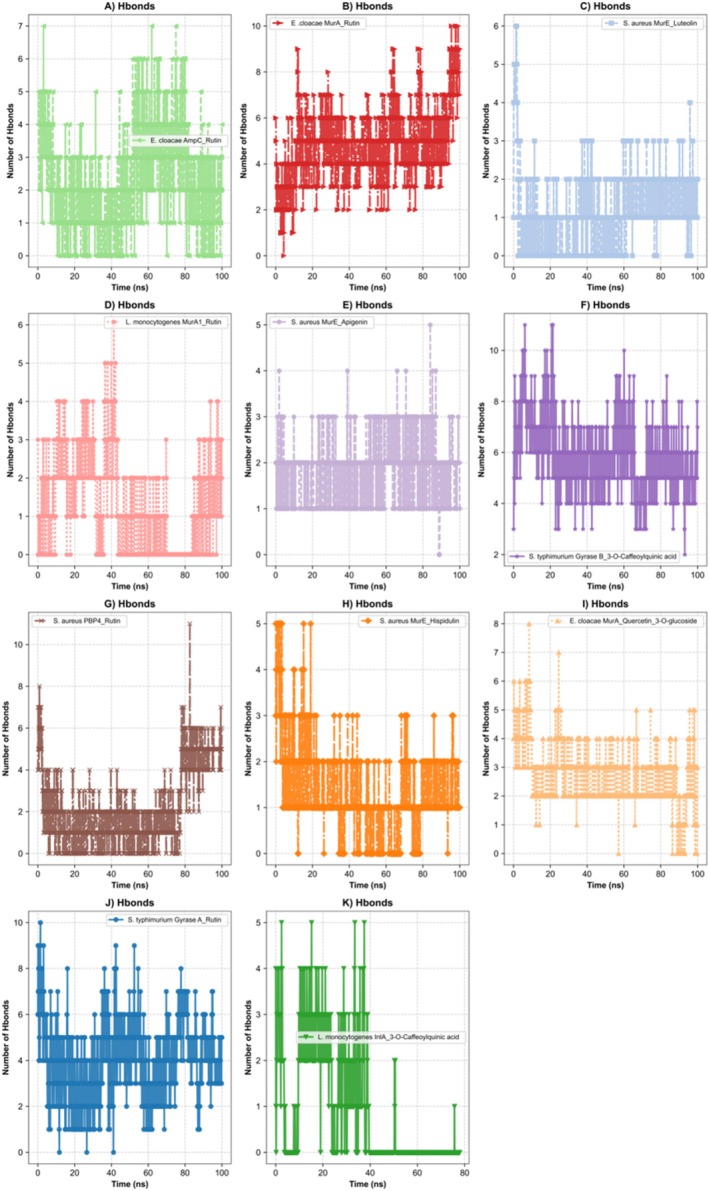
Hydrogen bond analysis. (A) Hydrogen bonds of the *E. cloacae* AmpC_Rutin complex. (B) Hydrogen bonds of the *E. cloacae* MurA_Rutin complex. (C) Hydrogen bonds of the *S. aureus* MurE_Luteolin complex. (D) Hydrogen bonds of the *L. monocytogenes* MurA1_Rutin complex. (E) Hydrogen bonds of the *S. aureus* MurE_Apigenin complex. (F) Hydrogen bonds of the *S. typhimurium* Gyrase B_3‐O‐Caffeoylquinic acid complex. (G) Hydrogen bonds of the *S. aureus* PBP4_Rutin complex. (H) Hydrogen bonds of the *S. aureus* MurE_Hispidulin complex. (I) Hydrogen bonds of the *E. cloacae* MurA_Quercetin_3‐O‐glucoside complex. (J) Hydrogen bonds of the *S. typhimurium* Gyrase A_Rutin complex. (K) Hydrogen bonds of the *L. monocytogenes* InlA_3‐O‐Caffeoylquinic acid complex.

Hydrogen bond analysis was used to assess interaction strength and persistence at the molecular level. The highest average number of hydrogen bonds was observed in *S. typhimurium* Gyrase B_3‐O‐Caffeoylquinic acid (Figure 3F) (5.89) and *E. cloacae* MurA_Rutin (Figure 3B) (4.83), indicating robust and stable binding. Although *S. typhimurium* Gyrase A_Rutin (Figure 3J) initially formed multiple hydrogen bonds, a progressive decline was detected. Reductions in hydrogen bonding were also apparent in *S. aureus* MurE_Hispidulin (Figure 3H), *L. monocytogenes* MurA1_Rutin (Figure 3D), and InlA_3‐O‐Caffeoylquinic acid (Figure 3K), reflecting weaker interaction stability. The *S. aureus* MurE_Apigenin (Figure 3E) complex maintained consistent hydrogen bonding, whereas MurE_Luteoli (Figure 3C) and PBP4_Rutin (Figure 3G) showed fluctuating profiles. In *E. cloacae* , both MurA_Quercetin‐3‐O‐glucoside (Figure 3I) and AmpC_Rutin (Figure 3A) exhibited declining Hbond trends over time. In summary, *S. typhimurium* Gyrase B_3‐O‐Caffeoylquinic acid (Figure 3F) and *E. cloacae* MurA–Rutin (Figure 3B) demonstrated the strongest and most persistent interactions, while *L. monocytogenes* MurA1–Rutin (Figure 3D) and InlA–3‐O‐Caffeoylquinic acid (Figure 3K) were characterized by weak and unstable binding.

**FIGURE 4:**
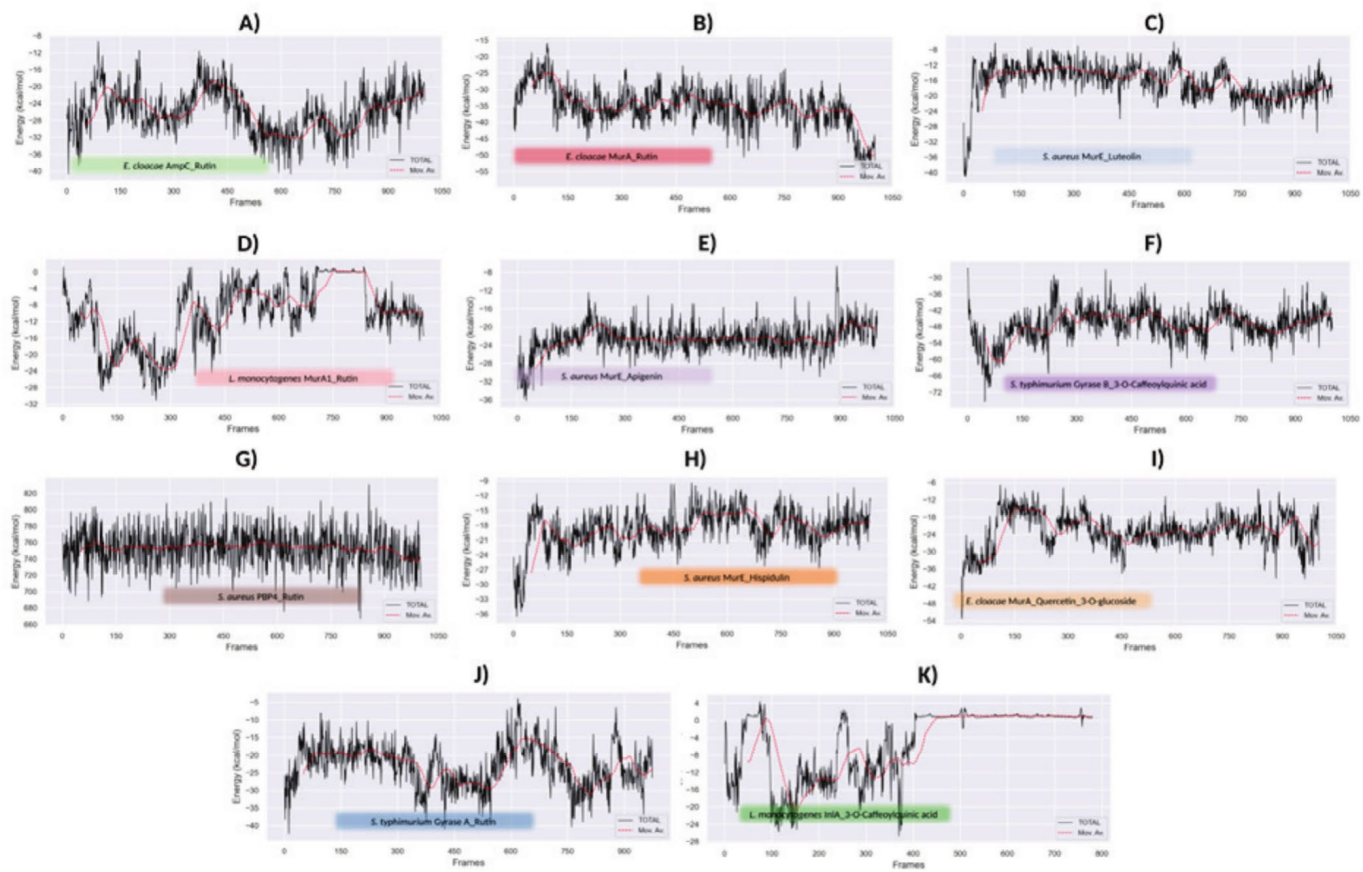
MM/PBSA analysis. (A) *E. cloacae* AmpC_Rutin complex. (B) *E. cloacae* MurA_Rutin complex. (C) *S. aureus* MurE_Luteolin complex. (D) *L. monocytogenes* MurA1_Rutin complex. (E) *S. aureus* MurE_Apigenin complex. (F) *S. typhimurium* Gyrase B_3‐O‐Caffeoylquinic acid complex. (G) *S. aureus* PBP4_Rutin complex. (H) *S. aureus* MurE_Hispidulin complex. (I) *E. cloacae* MurA_Quercetin_3‐O‐glucoside complex. (J) *S. typhimurium* Gyrase A_Rutin complex. (K) *L. monocytogenes* InlA_3‐O‐Caffeoylquinic acid complex.

Among the investigated systems, the *S. typhimurium* Gyrase B_3‐O‐Caffeoylquinic acid (Figure 4F), *E. cloacae* MurA_Rutin (Figure 4B), and *S. aureus* MurE_Apigenin (Figure 4E) exhibited highly stable binding profiles with sufficient simulation times. In contrast, although *E. cloacae* MurA_Quercetin‐3‐O‐glucoside (Figure 4I), *S. aureus* MurE_Hispidulin (Figure 4H), and *S. aureus* MurE_Luteolin (Figure 4C) also demonstrated favorable dynamic behaviors, extending the simulation time might provide a more comprehensive understanding of their long‐term stability. Meanwhile, *S. typhimurium* Gyrase A_Rutin (Figure 4J) showed acceptable stability despite energy fluctuations. Conversely, in the remaining complexes, including *L. monocytogenes* MurA1_Rutin (Figure 4D), *L. monocytogenes* InlA_3‐O‐Caffeoylquinic acid (Figure 4K), *S. aureus* PBP4_Rutin (Figure 4G), and *E. cloacae* AmpC_Rutin (Figure 4A), partial ligand displacement or unbinding events were detected, leading to the early termination of the simulations around 80 ns (Figure 2).

### 3.10 Free Energy Calculation Result

Following the MD simulation‐based evaluation of the studied systems, MM/PBSA calculations were performed to further validate the binding potentials of the 11 selected protein–ligand complexes over 100 ns trajectories. The selection of these complexes was based on their dynamic performance observed in the previous analyses (Figures 2 and 3), and their free energy profiles were interpreted within this context.

**FIGURE 5:**
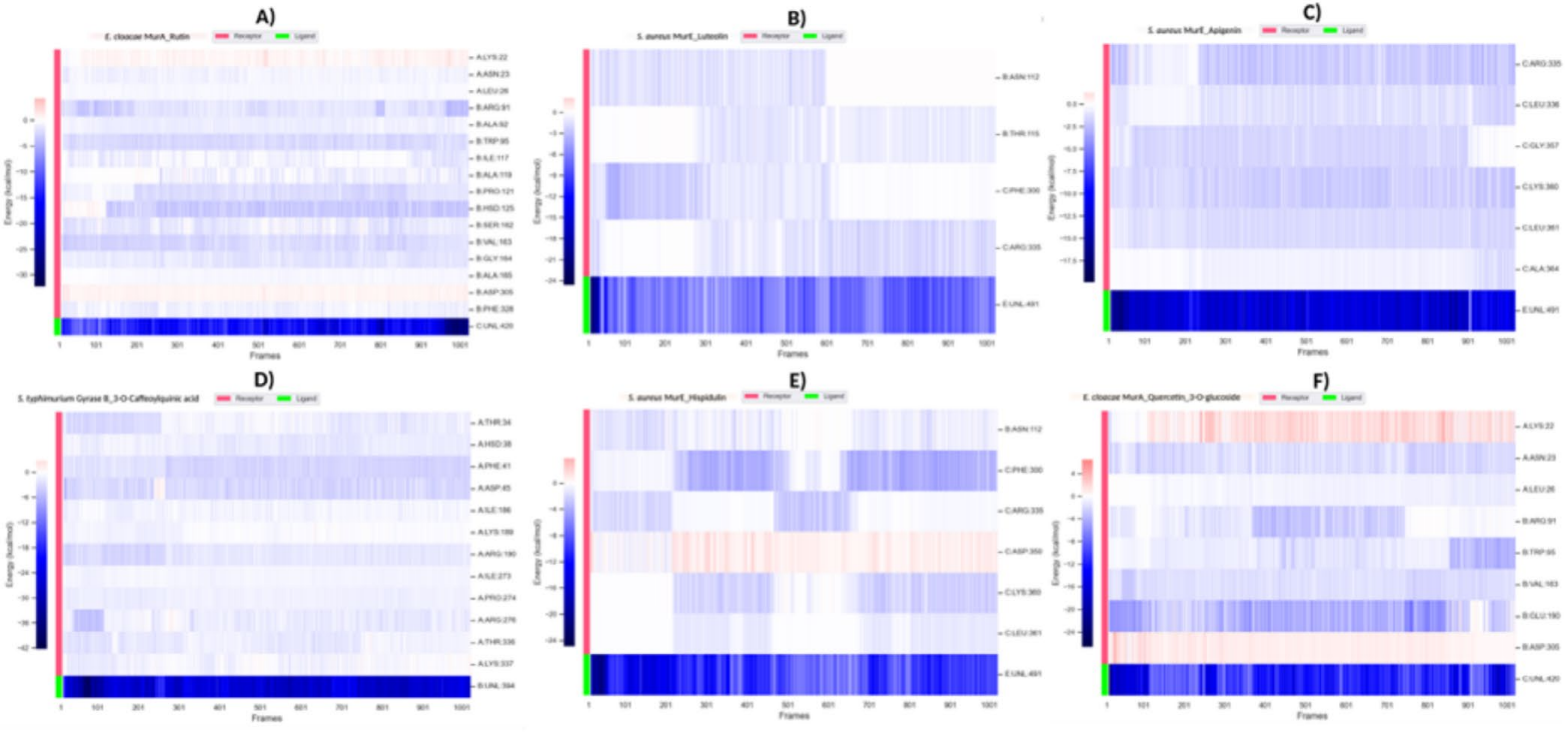
Binding free energy profiles of the best‐performing and time‐extension‐recommended complexes calculated using the MM/PBSA method over 100 ns simulation trajectories. The figure represents the total binding free energy variations (ΔGbind) of six selected protein–ligand complexes, highlighting their interaction stability and fluctuation patterns throughout the simulations. (A) *E. cloacae* MurA_Rutin, (B) *S. aureus* MurE_Luteolin, (C) *S. aureus* MurE_Apigenin, (D) *S. typhimurium* Gyrase B_3‐O‐Caffeoylquinic acid, (E) *S. aureus* MurE_Hispidulin, and (F) *E. cloacae* MurA_Quercetin‐3‐O‐glucoside.

In line with the MD results, *S. typhimurium Gyrase B and 3‐O‐Caffeoylquinic acid* (Figures 2, 3F, and 4F), *E. cloacae* MurA_Rutin (Figures 2, 3B, and 4B), and *S. aureus* MurE_Apigenin (Figures 2, 3E, and 4E), which exhibited highly stable dynamic behaviors during simulations, also demonstrated the most favorable binding energy profiles in MM/PBSA analysis, characterized by low energy values and minimal fluctuations. In these complexes, ligand binding was stabilized by multiple critical residues. Specifically, the *S. typhimurium* Gyrase B and 3‐O‐Caffeoylquinic acid interactions were supported by *THR34, HSD38, PHE41, ASP45, ILE186, LYS189, ARG190, ILE273, PRO274, ARG276, THR336, and LYS337* (Figure 5D). For the *E. cloacae* MurA and rutin, key residues involved in the binding were *LYS22, ASN23, LEU26, ARG91, ALA92, TRP95, ILE117, ALA119, PRO121, HSD125, SER162, VAL163, GLY164, ALA165, ASP305, and PHE328* (Figure 5A). Likewise, *E. cloacae* MurE and Apigenin binding was stabilized by *ARG335, LEU336, GLY357, LYS360, LEU361, and ALA364* (Figure 5C).

Similarly, *E. cloacae* MurA_Quercetin‐3‐O‐glucoside (Figures 2, 3I, and 4I), *S. aureus* MurE_Hispidulin (Figures 2, 3H, and 4H), and *S. aureus* MurE_Luteolin (Figures 2, 3C, and 4C), which were categorized as dynamically stable but recommended for extended simulation periods, also displayed energetically stable binding profiles with relatively low fluctuation patterns, supporting their interaction potential. The binding of the *E. cloacae* MurA_Quercetin‐3‐O‐glucoside complex was mediated by *LYS22, ASN23, LEU26, ARG91, ALA92, TRP95, ILE117, ALA119, PRO121, HSD125, SER162, VAL163, GLY164, ALA165, ASP305*, and *PHE328* (Figure 5F). In the *S. aureus* MurE_Hispidulin complex, residues *ASN112, PHE300, ARG335, ASP350, LYS360, and LEU361* played key roles (Figure 5E). Additionally, *S. aureus* MurE and luteolin interactions were stabilized by *ASN112, THR115, PHE300, and ARG335* (Figure 5B).

Conversely, *L. monocytogenes* MurA1*_*Rutin (Figures 2, 3D, and 4D), *L. monocytogenes* InlA_3‐O‐Caffeoylquinic acid (Figures 2, 3K, and 4K), *S. aureus* PBP4_Rutin (Figures 2, 3G, and 4G), and *E. cloacae* AmpC_Rutin (Figures 2A, 3A, and 4A), complexes that had shown lower stability or ligand displacement in MD simulations, presented higher binding energy values and more pronounced fluctuation profiles, further supporting their relatively weaker binding stability.

## 4 Conclusions

Native to Turkmenistan, *Ziziphora galinae* extracts have displayed notable bioactivity across diverse assays. The 70% ethanol extract demonstrated exceptional antioxidant capacity and enzyme inhibition, linked to its higher phenolic and flavonoid content. Both extracts showed wide‐ranging antimicrobial effects, with stronger antibacterial than antifungal efficacy. Molecular docking indicated stable interactions between main phytochemicals (rutin, apigenin) and bacterial/human enzyme targets, implying potential dual inhibition. Although the extracts exhibited limited cytotoxicity on HaCaT cells, their broad bioactivity highlights therapeutic promise, especially for neurodegenerative, metabolic, and infectious diseases. This research offers the first detailed phytochemical analysis of ZG, laying groundwork for further pharmacological advancement.

## Conflicts of Interest

The authors declare no conflicts of interest.

## Data Availability

Data will be made available on request.

## References

[fsn370875-bib-0001] Aazza, S. , B. Lyoussi , and M. G. Miguel . 2011. “Antioxidant and Antiacetylcholinesterase Activities of Some Commercial Essential Oils and Their Major Compounds.” Molecules 16: 7672–7690. 21900869 10.3390/molecules16097672 PMC6264425

[fsn370875-bib-0002] Abubakar, A. R. , and M. Haque . 2020. “Preparation of Medicinal Plants: Basic Extraction and Fractionation Procedures for Experimental Purposes.” Journal of Pharmacy & Bioallied Sciences 12: 1–10. 32801594 10.4103/jpbs.JPBS_175_19 PMC7398001

[fsn370875-bib-0003] Acosta‐Gutiérrez, S. , L. Ferrara , M. Pathania , et al. 2018. “Getting Drugs Into Gram‐Negative Bacteria: Rational Rules for Permeation Through General Porins.” ACS Infectious Diseases 4: 1487–1498. 29962203 10.1021/acsinfecdis.8b00108

[fsn370875-bib-0004] Agafonova, A. , T. Bagaeva , V. Artemieva , T. Yamashev , and O. Reshetnik . 2019. “Influence of the Solvent on the Antioxidant Properties of Extracts.” Helix 9: 5312.

[fsn370875-bib-0005] Alexander, J. A. N. , S. S. Chatterjee , S. M. Hamilton , L. D. Eltis , H. F. Chambers , and N. C. Strynadka . 2018. “Structural and Kinetic Analyses of Penicillin‐Binding Protein 4 (PBP4)‐mediated Antibiotic Resistance in *Staphylococcus Aureus* .” Journal of Biological Chemistry 293: 19854–19865. 30366985 10.1074/jbc.RA118.004952 PMC6314119

[fsn370875-bib-0006] Apak, R. , K. Güçlü , M. Özyürek , and S. E. Karademir . 2004. “Novel Total Antioxidant Capacity Index for Dietary Polyphenols and Vitamins C and E, Using Their Cupric Ion Reducing Capability in the Presence of Neocuproine: CUPRAC Method.” Journal of Agricultural and Food Chemistry 52: 7970–7981. 15612784 10.1021/jf048741x

[fsn370875-bib-0007] Aqil, F. , M. Zahin , I. Ahmad , et al. 2010. “Antifungal Activity of Medicinal Plant Extracts and Phytocompounds: A Review.” In Combating Fungal Infections: Problems and Remedy, 449–484. Springer.

[fsn370875-bib-0008] Aryal, S. , M. K. Baniya , K. Danekhu , P. Kunwar , R. Gurung , and N. Koirala . 2019. “Total Phenolic Content, Flavonoid Content and Antioxidant Potential of Wild Vegetables From Western Nepal.” Plants 8: 96. 30978964 10.3390/plants8040096 PMC6524357

[fsn370875-bib-0009] Bahadori, M. B. , B. Kirkan , and C. Sarikurkcu . 2019. “Phenolic Ingredients and Therapeutic Potential of *Stachys cretica* Subsp. Smyrnaea for the Management of Oxidative Stress, Alzheimer's Disease, Hyperglycemia, and Melasma.” Industrial Crops and Products 127: 82–87.

[fsn370875-bib-0010] Barna, T. M. , H. Khan , N. C. Bruce , I. Barsukov , N. S. Scrutton , and P. C. Moody . 2001. “Crystal Structure of Pentaerythritol Tetranitrate Reductase: “Flipped” Binding Geometries for Steroid Substrates in Different Redox States of the Enzyme.” Journal of Molecular Biology 310: 433–447. 11428899 10.1006/jmbi.2001.4779

[fsn370875-bib-0011] Belousoff, M. J. , Z. Eyal , M. Radjainia , et al. 2017. “Structural Basis for Linezolid Binding Site Rearrangement in the *Staphylococcus aureus* Ribosome.” MBio 8: e00395‐17. 10.1128/mbio.00395-00317. 28487427 PMC5424203

[fsn370875-bib-0012] Belwal, T. , S. M. Ezzat , L. Rastrelli , et al. 2018. “A Critical Analysis of Extraction Techniques Used for Botanicals: Trends, Priorities, Industrial Uses and Optimization Strategies.” TrAC Trends in Analytical Chemistry 100: 82–102. 10.1016/j.trac.2017.12.018.

[fsn370875-bib-0013] Benzie, I. F. , and J. J. Strain . 1996. “The Ferric Reducing Ability of Plasma (FRAP) as a Measure of “Antioxidant Power”: The FRAP Assay.” Analytical Biochemistry 239: 70–76. 8660627 10.1006/abio.1996.0292

[fsn370875-bib-0014] Berdimuhamedow, G. 2010. Türkmenistanyň Dermanlyk Ösümlikleri. Türkmen Döwlet Neşirýat Gullugy.

[fsn370875-bib-0015] Bhebhe, M. , T. N. Füller , B. Chipurura , and M. Muchuweti . 2016. “Effect of Solvent Type on Total Phenolic Content and Free Radical Scavenging Activity of Black Tea and Herbal Infusions.” Food Analytical Methods 9: 1060–1067.

[fsn370875-bib-0016] Brighente, I. , M. Dias , L. Verdi , and M. Pizzolatti . 2007. “Antioxidant Activity and Total Phenolic Content of Some Brazilian Species.” Pharmaceutical Biology 45: 156–161.

[fsn370875-bib-0017] Bublitz, M. , C. Holland , C. Sabet , et al. 2008. “Crystal Structure and Standardized Geometric Analysis of InlJ, a Listerial Virulence Factor and Leucine‐Rich Repeat Protein With a Novel Cysteine Ladder.” Journal of Molecular Biology 378: 87–96. 18343406 10.1016/j.jmb.2008.01.100

[fsn370875-bib-0018] Canga, I. , P. Vita , A. I. Oliveira , M. Á. Castro , and C. Pinho . 2022. “In Vitro Cytotoxic Activity of African Plants: A Review.” Molecules 27: 4989. 35956938 10.3390/molecules27154989 PMC9370645

[fsn370875-bib-0019] Ćavar Zeljković, S. , E. Schadich , P. Džubák , M. Hajdúch , and P. Tarkowski . 2022. “Antiviral Activity of Selected Lamiaceae Essential Oils and Their Monoterpenes Against SARS‐CoV‐2.” Frontiers in Pharmacology 13: 893634. 35586050 10.3389/fphar.2022.893634 PMC9108200

[fsn370875-bib-0092] Cordeiro, N. , A. C. Pinguelli Ristau , G. Costa Braga , D. Fernandes da Silva , F. Villa , and E. Soaresde Vasconcelos . 2022. “Extraction of Antioxidant Compounds From Dovyalis: Mixtures of Ethanol and Water.” Scientia Agraria Paranaensis 21, no. 3: 300.

[fsn370875-bib-0020] Daouk, R. K. , S. M. Dagher , and E. J. Sattout . 1995. “Antifungal Activity of the Essential Oil of *Origanum syriacum* L.” Journal of Food Protection 58: 1147–1149. 31137364 10.4315/0362-028X-58.10.1147

[fsn370875-bib-0021] Dileep, K. V. , K. Ihara , C. Mishima‐Tsumagari , et al. 2022. “Crystal Structure of Human Acetylcholinesterase in Complex With Tacrine: Implications for Drug Discovery.” International Journal of Biological Macromolecules 210: 172–181. 10.1016/j.ijbiomac.2022.05.009. 35526766

[fsn370875-bib-0022] Dinis, T. C. , V. M. Madeira , and L. M. Almeida . 1994. “Action of Phenolic Derivatives (Acetaminophen, Salicylate, and 5‐Aminosalicylate) as Inhibitors of Membrane Lipid Peroxidation and as Peroxyl Radical Scavengers.” Archives of Biochemistry and Biophysics 315: 161–169. 7979394 10.1006/abbi.1994.1485

[fsn370875-bib-0089] Dudonne, S. , X. Vitrac , P. Coutiere , M. Woillez , and J. M. Mérillon . 2009. “Comparative Study of Antioxidant Properties and Total Phenolic Content of 30 Plant Extracts of Industrial Interest Using DPPH, ABTS, FRAP, SOD, and ORAC Assays.” Journal of Agricultural and Food Chemistry 57, no. 5: 1768–1774. 19199445 10.1021/jf803011r

[fsn370875-bib-0023] Ellman, G. L. , K. D. Courtney , V. Andres Jr. , and R. M. Featherstone . 1961. “A New and Rapid Colorimetric Determination of Acetylcholinesterase Activity.” Biochemical Pharmacology 7: 88–95. 13726518 10.1016/0006-2952(61)90145-9

[fsn370875-bib-0093] Galanakis, C. M. , V. Goulas , S. Tsakona , G. A. Manganaris , and V. Gekas . 2013. “A Knowledge Base for the Recovery of Natural Phenols With Different Solvents.” International Journal of Food Properties 16, no. 2: 382–396.

[fsn370875-bib-0024] Gomes, B. , C. Ferraz , V. Me , V. Berber , F. Teixeira , and F. Souza‐Filho . 2001. “In Vitro Antimicrobial Activity of Several Concentrations of Sodium Hypochlorite and Chlorhexidine Gluconate in the Elimination of *Enterococcus Faecalis* .” International Endodontic Journal 34: 424–428. 11556507 10.1046/j.1365-2591.2001.00410.x

[fsn370875-bib-0025] Greig, N. H. , D. K. Lahiri , and K. Sambamurti . 2002. “Butyrylcholinesterase: An Important New Target in Alzheimer's Disease Therapy.” International Psychogeriatrics 14: 77–91. 12636181 10.1017/s1041610203008676

[fsn370875-bib-0026] Greta, U. , G. Hasmik , D. Karine , et al. 2022. “The Study of the Biological Activities of Ziziphora Clinopodioides.” Brazilian Journal of Pharmaceutical Sciences 58: e19331.

[fsn370875-bib-0027] Gupta, D. , P. Tiwari , M. A. Haque , et al. 2021. “Structural Insights Into the Transient Closed Conformation and pH Dependent ATPase Activity of *S. typhi* GyraseB N‐Terminal Domain.” Archives of Biochemistry and Biophysics 701: 108786. 33548211 10.1016/j.abb.2021.108786

[fsn370875-bib-0028] Hampele, I. C. , A. D'Arcy , G. E. Dale , et al. 1997. “Structure and Function of the Dihydropteroate Synthase From *Staphylococcus aureus* .” Elsevier 268: 21–30. 10.1006/jmbi.1997.0944 9149138

[fsn370875-bib-0029] Han, H. , Y. Yang , S. H. Olesen , A. Becker , S. Betzi , and E. Schönbrunn . 2010. “The Fungal Product Terreic Acid Is a Covalent Inhibitor of the Bacterial Cell Wall Biosynthetic Enzyme UDP‐N‐Acetylglucosamine 1‐Carboxyvinyltransferase (MurA).” Biochemistry 49: 4276–4282. 20392080 10.1021/bi100365b PMC2884014

[fsn370875-bib-0030] Haynes, C. A. , R. L. Koder , A.‐F. Miller , and D. W. Rodgers . 2002. “Structures of Nitroreductase in Three States: Effects of Inhibitor Binding and Reduction.” Journal of Biological Chemistry 277: 11513–11520. 11805110 10.1074/jbc.M111334200

[fsn370875-bib-0031] Ilhan, M. , P. Gürbüz , and İ. Süntar . 2025. “An Updated Review on *Ziziphora* L.: A Valuable Source of Phytoconstituents for Potential Health Benefits.” Records of Natural Products 19: 375–399.

[fsn370875-bib-0032] Karimifar, P. , S. S. Saei‐Dehkordi , and Z. Izadi . 2022. “Antibacterial, Antioxidative and Sensory Properties of Ziziphora Clinopodioides– *Rosmarinus officinalis* Essential Oil Nanoencapsulated Using Sodium Alginate in Raw Lamb Burger Patties.” Food Bioscience 47: 101698.

[fsn370875-bib-0033] Kawai, A. , C. L. McElheny , A. Iovleva , et al. 2020. “Structural Basis of Reduced Susceptibility to Ceftazidime‐Avibactam and Cefiderocol in *Enterobacter Cloacae* due to AmpC R2 Loop Deletion.” Antimicrobial Agents and Chemotherapy 64: 10‐1128. 10.1128/aac.00198-00120. PMC7318025 32284381

[fsn370875-bib-0034] Kirby, A. J. , and R. J. Schmidt . 1997. “The Antioxidant Activity of Chinese Herbs for Eczema and of Placebo Herbs—I.” Journal of Ethnopharmacology 56: 103–108. 9174970 10.1016/s0378-8741(97)01510-9

[fsn370875-bib-0035] Kong, L. , S.‐J. Park , and W. Im . 2024. “CHARMM‐GUI PDB Reader and Manipulator: Covalent Ligand Modeling and Simulation.” Journal of Molecular Biology 436: 168554. 39237201 10.1016/j.jmb.2024.168554 PMC11377865

[fsn370875-bib-0036] Konyalιoglu, S. , B. Ozturk , and G. E. Meral . 2006. “Comparison of Chemical Compositions and Antioxidant Activities of the Essential Oils of Two Ziziphora. Taxa From Anatolia.” Pharmaceutical Biology 44: 121–126.

[fsn370875-bib-0037] Köster, S. , K. Van Pee , M. Hudel , et al. 2014. “Crystal Structure of Listeriolysin O Reveals Molecular Details of Oligomerization and Pore Formation.” Nature Communications 5: 3690. 10.1038/ncomms4690 24751541

[fsn370875-bib-0038] Kowalska‐Krochmal, B. , and R. Dudek‐Wicher . 2021. “The Minimum Inhibitory Concentration of Antibiotics: Methods, Interpretation, Clinical Relevance.” Pathogens 10: 165. 33557078 10.3390/pathogens10020165 PMC7913839

[fsn370875-bib-0039] Kulen, M. , M. Lindgren , S. Hansen , et al. 2018. “Structure‐Based Design of Inhibitors Targeting PrfA, the Master Virulence Regulator of *Listeria Monocytogenes* .” Journal of Medicinal Chemistry 61: 4165–4175. 29667825 10.1021/acs.jmedchem.8b00289

[fsn370875-bib-0040] Lambert, R. , P. N. Skandamis , P. J. Coote , and G. J. Nychas . 2001. “A Study of the Minimum Inhibitory Concentration and Mode of Action of Oregano Essential Oil, Thymol and Carvacrol.” Journal of Applied Microbiology 91: 453–462. 11556910 10.1046/j.1365-2672.2001.01428.x

[fsn370875-bib-0042] Lu, J. , S. Patel , N. Sharma , et al. 2014. “Structures of Kibdelomycin Bound to *Staphylococcus aureus* GyrB and ParE Showed a Novel U‐Shaped Binding Mode.” ACS Chemical Biology 9: 2023–2031. 24992706 10.1021/cb5001197

[fsn370875-bib-0043] Lund, B. A. , A. M. Thomassen , T. J. W. Carlsen , and H.‐K. Leiros . 2021. “Biochemical and Biophysical Characterization of the OXA‐48‐Like Carbapenemase OXA‐436.” Acta Crystallographica Section F: Structural Biology Communications 77: 312–318. 34473108 10.1107/S2053230X21008645 PMC8411929

[fsn370875-bib-0044] Maier, J. A. , C. Martinez , K. Kasavajhala , L. Wickstrom , K. E. Hauser , and C. Simmerling . 2015. “ff14SB: Improving the Accuracy of Protein Side Chain and Backbone Parameters From ff99SB.” Journal of Chemical Theory and Computation 11: 3696–3713. 26574453 10.1021/acs.jctc.5b00255 PMC4821407

[fsn370875-bib-0045] Malaník, M. , J. Treml , R. Kubínová , et al. 2025. “Isolation of a Unique Monoterpene Diperoxy Dimer From Ziziphora Clinopodioides Subsp. Bungeana Together With Triterpenes With Antidiabetic Properties.” Phytochemical Analysis 36: 1223–1230. 39780359 10.1002/pca.3505 PMC12129716

[fsn370875-bib-0046] Masuda, T. , D. Yamashita , Y. Takeda , and S. Yonemori . 2005. “Screening for Tyrosinase Inhibitors Among Extracts of Seashore Plants and Identification of Potent Inhibitors From Garcinia Subelliptica.” Bioscience, Biotechnology, and Biochemistry 69: 197–201. 15665485 10.1271/bbb.69.197

[fsn370875-bib-0047] Mervin, L. H. , Q. Cao , I. P. Barrett , et al. 2016. “Understanding Cytotoxicity and Cytostaticity in a High‐Throughput Screening Collection.” ACS Chemical Biology 11: 3007–3023. 27571164 10.1021/acschembio.6b00538

[fsn370875-bib-0048] Miller, B. R., III , T. D. McGee Jr. , J. M. Swails , N. Homeyer , H. Gohlke , and A. E. Roitberg . 2012. “MMPBSA. Py: An Efficient Program for End‐State Free Energy Calculations.” Journal of Chemical Theory and Computation 8: 3314–3321. 26605738 10.1021/ct300418h

[fsn370875-bib-0049] Moghadam, H. D. , A. M. Sani , and M. M. Sangatash . 2016. “Antifungal Activity of Essential Oil of Ziziphora Clinopodioides and the Inhibition of Aflatoxin B1 Production in Maize Grain.” Toxicology and Industrial Health 32: 493–499. 24193054 10.1177/0748233713503375

[fsn370875-bib-0050] Mohammadhosseini, M. 2017. “The Ethnobotanical, Phytochemical and Pharmacological Properties and Medicinal Applications of Essential Oils and Extracts of Different Ziziphora Species.” Industrial Crops and Products 105: 164–192.

[fsn370875-bib-0051] Moser, J. , B. Gerstel , J. E. Meyer , T. Chakraborty , J. Wehland , and D. W. Heinz . 1997. “Crystal Structure of the Phosphatidylinositol‐Specific Phospholipase C From the Human Pathogen *Listeria monocytogenes* .” Journal of Molecular Biology 273: 269–282. 9367761 10.1006/jmbi.1997.1290

[fsn370875-bib-0090] Mutha, R. E. , A. U. Tatiya , and S. J. Surana . 2021. “Flavonoids as Natural Phenolic Compounds and Their Role in Therapeutics: An Overview.” Future Journal of Pharmaceutical Sciences 7, no. 1: 25. 33495733 10.1186/s43094-020-00161-8 PMC7816146

[fsn370875-bib-0052] Nikitin, V. V. E. , and A. M. Gel'dikhanov . 1988. “Opredelitel' rasteniĭ Turkmenistana. ‘Nauka’, Leningradskoe otd‐nie”.

[fsn370875-bib-0053] Ooi, A. , S. Hussain , A. Seyedarabi , and R. W. Pickersgill . 2006. “Structure of Internalin C From *Listeria Monocytogenes* .” Acta Crystallographica Section D: Biological Crystallography 62: 1287–1293. 17057330 10.1107/S0907444906026746

[fsn370875-bib-0054] Ordaz‐Hernández, A. , B. Hernández‐Carlos , H. M. A. González , L. Hernández‐Ramiro , M. C. Ramírez , and M. Herrera‐Martínez . 2024. “ *Parkinsonia praecox* Bark as a New Source of Antibacterial and Anticancer Compounds.” European Journal of Integrative Medicine 71: 102401. 10.1016/j.eujim.2024.102401.

[fsn370875-bib-0055] Owens, T. W. , R. J. Taylor , K. S. Pahil , et al. 2019. “Structural Basis of Unidirectional Export of Lipopolysaccharide to the Cell Surface.” Nature 567: 550–553. 30894747 10.1038/s41586-019-1039-0 PMC6629255

[fsn370875-bib-0056] Özkan, E. E. , M. Boğa , M. A. Yılmaz , E. M. Kara , and Y. Yeşil . 2020. “LC‐MS/MS Analyses of Ziziphora Clinopodioides Lam. From Turkey: Antioxidant, Anticholinesterase, Antimicrobial and, Anticancer Activities.” İstanbul Journal of Pharmacy 50: 33–41.

[fsn370875-bib-0057] Ozturk, S. , and S. Ercisli . 2007. “Antibacterial Activity and Chemical Constitutions of Ziziphora Clinopodioides.” Food Control 18: 535–540.

[fsn370875-bib-0058] Palombo, E. A. 2011. “Traditional Medicinal Plant Extracts and Natural Products With Activity Against Oral Bacteria: Potential Application in the Prevention and Treatment of Oral Diseases.” Evidence‐based Complementary and Alternative Medicine 2011: 680354. 19596745 10.1093/ecam/nep067 PMC3145422

[fsn370875-bib-0059] Pane, Y. S. 2024. “Effectiveness of Traditional Herbal Extracts Against Multidrug‐Resistant Bacteria: A Review.” 2024.2011.2003.621775.

[fsn370875-bib-0041] Plant List . 2024. “Plants of the World Online | Kew Science., ’Ziziphora galinae Juz’.” 2024.

[fsn370875-bib-0060] Prieto, P. , M. Pineda , and M. Aguilar . 1999. “Spectrophotometric Quantitation of Antioxidant Capacity Through the Formation of a Phosphomolybdenum Complex: Specific Application to the Determination of Vitamin E.” Analytical Biochemistry 269: 337–341. 10222007 10.1006/abio.1999.4019

[fsn370875-bib-0061] Qader, O. , M. A.‐S. Saa , and D. Al‐Fekaiki . 2023. “Antibacterial and Antioxidant Activity of Ziziphora Clinopodioid Lam.(Lamiaceae) Essential Oil.” Archives of Razi Institute 78: 205. 37312711 10.22092/ARI.2022.358487.2228 PMC10258302

[fsn370875-bib-0062] Quettier‐Deleu, C. , B. Gressier , J. Vasseur , et al. 2000. “Phenolic Compounds and Antioxidant Activities of Buckwheat ( *Fagopyrum esculentum* Moench) Hulls and Flour.” Journal of Ethnopharmacology 72: 35–42. 10.1016/S0378-8741(00)00196-3. 10967451

[fsn370875-bib-0063] Radović, M. , D. Milatović , Ž. Tešić , et al. 2020. “Influence of Rootstocks on the Chemical Composition of the Fruits of Plum Cultivars.” Journal of Food Composition and Analysis 92: 103480.

[fsn370875-bib-0064] Re, R. , N. Pellegrini , A. Proteggente , A. Pannala , M. Yang , and C. Rice‐Evans . 1999. “Antioxidant Activity Applying an Improved ABTS Radical Cation Decolorization Assay.” Free Radical Biology and Medicine 26: 1231–1237. 10381194 10.1016/s0891-5849(98)00315-3

[fsn370875-bib-0065] Rosenberry, T. L. , X. Brazzolotto , I. R. Macdonald , et al. 2017. “Comparison of the Binding of Reversible Inhibitors to Human Butyrylcholinesterase and Acetylcholinesterase: A Crystallographic, Kinetic and Calorimetric Study.” Molecules 22, no. 10: 3390/molecules22122098. 10.3390/molecules22122098 PMC6149722 29186056

[fsn370875-bib-0091] Roy, A. , A. Khan , I. Ahmad , et al. 2022. “Flavonoids a Bioactive Compound From Medicinal Plants and its Therapeutic Applications.” BioMed Research International 2022, no. 1: 5445291. 35707379 10.1155/2022/5445291 PMC9192232

[fsn370875-bib-0066] Sachdeva, E. , G. Kaur , P. Tiwari , et al. 2020. “The Pivot Point Arginines Identified in the β‐Pinwheel Structure of C‐Terminal Domain From *Salmonella Typhi* DNA Gyrase A Subunit.” Scientific Reports 10: 7817. 32385379 10.1038/s41598-020-64792-w PMC7210945

[fsn370875-bib-0067] Šafašík, I. 1990. “Rapid Detection of Alpha‐Amylase Inhibitors.” Journal of Enzyme Inhibition 3: 245–247. 2079642 10.3109/14756369009035843

[fsn370875-bib-0068] Salehi, P. , A. Sonboli , F. Eftekhar , S. Nejad‐Ebrahimi , and M. Yousefzadi . 2005. “Essential Oil Composition, Antibacterial and Antioxidant Activity of the Oil and Various Extracts of Ziziphora Clinopodioides Subsp. Rigida (B OISS.) R ECH. f. From Iran.” Biological and Pharmaceutical Bulletin 28: 1892–1896. 16204941 10.1248/bpb.28.1892

[fsn370875-bib-0069] Sarikurkcu, C. , E. Kakouri , R. T. Sarikurkcu , and P. A. Tarantilis . 2019. “Study on the Chemical Composition, Enzyme Inhibition and Antioxidant Activity of Ziziphora Taurica Subsp. Cleonioides.” Applied Sciences 9: 5515.

[fsn370875-bib-0070] Schubert, W.‐D. , C. Urbanke , T. Ziehm , et al. 2002. “Structure of Internalin, a Major Invasion Protein of *Listeria monocytogenes* , in Complex With Its Human Receptor E‐Cadherin.” Cell 111: 825–836. 12526809 10.1016/s0092-8674(02)01136-4

[fsn370875-bib-0071] Selvi, S. , and F. Satil . 2020. “Comparative Anatomy on the Vegetative Organs of Genus Ziziphora L.(Lamiaceae) From Turkey.” Microscopy Research and Technique 83: 10–21. 31617645 10.1002/jemt.23383

[fsn370875-bib-0072] Shahbazi, Y. 2017. “Chemical Compositions, Antioxidant and Antimicrobial Properties of Ziziphora Clinopodioides Lam. Essential Oils Collected From Different Parts of Iran.” Journal of Food Science and Technology 54: 3491–3503. 29051644 10.1007/s13197-017-2806-2 PMC5629158

[fsn370875-bib-0073] Sharopov, F. S. , and W. N. Setzer . 2011. “Chemical Diversity of Ziziphora Clinopodioides: Composition of the Essential Oil of Z. Clinopodioides From Tajikistan.” Natural Product Communications 6: 1934578X1100600524. 21615034

[fsn370875-bib-0074] Shomali, T. 2019. “Zataria Multiflora and Gastrointestinal Tract Disorders.” In Dietary Interventions in Gastrointestinal Diseases, 209–212. Elsevier.

[fsn370875-bib-0075] Sinaeyan, S. , and A. Sani . 2014. “Antimicrobial Activity of Ziziphora Clinopodioides Essential Oil and Extract on *Salmonella enterica* , Staphylococcus Aureus and *Saccharomyces cerevisiae* in Low Fat Mayonnaise.” BTAIJ 10: 24.

[fsn370875-bib-0076] Slinkard, K. , and V. L. Singleton . 1977. “Total Phenol Analysis: Automation and Comparison With Manual Methods.” American Journal of Enology and Viticulture 28: 49–55.

[fsn370875-bib-0077] Šmejkal, K. , M. Malaník , K. Zhaparkulova , et al. 2016. “Kazakh Ziziphora Species as Sources of Bioactive Substances.” Molecules 21: 826. 27347924 10.3390/molecules21070826 PMC6274025

[fsn370875-bib-0078] Sugiarti, L. , D. A. Palupi , and I. Febriana . 2022. “Effect of Ethanol Extract From Herbal Consortium for Pytirosporum Ovale Inhibition.” Sains Natural: Journal of Biology and Chemistry 12: 184–191.

[fsn370875-bib-0079] Swarén, P. , L. Maveyraud , X. Raquet , et al. 1998. “X‐Ray Analysis of the NMC‐A β‐Lactamase at 1.64‐Å Resolution, a Class A Carbapenemase With Broad Substrate Specificity.” Journal of Biological Chemistry 273: 26714–26721. 9756914 10.1074/jbc.273.41.26714

[fsn370875-bib-0080] Taheri, A. , A. Ganjeali , A. Arefi‐Oskouie , C. Çirak , and M. Cheniany . 2023. “The Variability of Phenolic Constituents and Antioxidant Properties Among Wild Populations of Ziziphora Clinopodioides Lam.” Physiology and Molecular Biology of Plants 29: 221–237. 36875730 10.1007/s12298-023-01283-y PMC9981857

[fsn370875-bib-0094] Tella, J. O. , and S. O. Oseni . 2019. “Comparative Profiling of Solvent‐Mediated Phytochemical Expressions in *Ocimum gratissimum* and *Vernonia amygdalina* Leaf Tissues via FTIR Spectroscopy and Colorimetric Assays.” Journal of Advances in Medical and Pharmaceutical Sciences 19: 1–25.

[fsn370875-bib-0081] Ting, L. , X.‐d. Zhang , Y.‐w. Song , and J.‐w. Liu . 2005. “A Microplate‐Based Screening Method for Alpha‐Glucosidase Inhibitors.” Chinese Journal of Clinical Pharmacology and Therapeutics 10: 1128.

[fsn370875-bib-0082] Tomczyk, M. , O. Ceylan , M. Locatelli , A. Tartaglia , V. Ferrone , and C. Sarikurkcu . 2019. “Ziziphora Taurica Subsp. Taurica: Analytical Characterization and Biological Activities.” Biomolecules 9: 367. 31416216 10.3390/biom9080367 PMC6723581

[fsn370875-bib-0083] Trott, O. , and A. J. Olson . 2010. “AutoDock Vina: Improving the Speed and Accuracy of Docking With a New Scoring Function, Efficient Optimization, and Multithreading.” Journal of Computational Chemistry 31: 455–461. 10.1002/jcc.21334. 19499576 PMC3041641

[fsn370875-bib-0084] Valdés‐Tresanco, M. S. , M. E. Valdés‐Tresanco , P. A. Valiente , and E. Moreno . 2021. “gmx_MMPBSA: A New Tool to Perform End‐State Free Energy Calculations With GROMACS.” Journal of Chemical Theory and Computation 17: 6281–6291. 34586825 10.1021/acs.jctc.1c00645

[fsn370875-bib-0085] Wang, L. , X. Shao , T. Zhong , et al. 2021. “Discovery of a First‐In‐Class CDK2 Selective Degrader for AML Differentiation Therapy.” Nature Chemical Biology 17: 567–575. 33664520 10.1038/s41589-021-00742-5

[fsn370875-bib-0086] Yamasaki, S. , R. Nakashima , K. Sakurai , et al. 2019. “Crystal Structure of the Multidrug Resistance Regulator RamR Complexed With Bile Acids.” Scientific Reports 9: 177. 30655545 10.1038/s41598-018-36025-8 PMC6336783

[fsn370875-bib-0087] Zhang, X. , W. Ding , J. Li , F. Liu , X. Zhou , and S. Tian . 2015. “Multi‐Elemental Analysis of Ziziphora Clinopodioides From Different Regions, Periods and Parts Using Atomic Absorption Spectrometry and Chemometric Approaches.” Revista Brasileira de Farmacognosia 25: 465–472.

[fsn370875-bib-0088] Zhaparkulova, K. , A. Karaubayeva , Z. Sakipova , et al. 2022. “Multidirectional Characterization of Phytochemical Profile and Health‐Promoting Effects of Ziziphora Bungeana Juz. Extracts.” Molecules 27: 8994. 36558125 10.3390/molecules27248994 PMC9788533

[fsn370875-bib-0095] Zolghadri, S. , A. Bahrami , M. T. Hassan Khan , et al. 2019. “A Comprehensive Review on Tyrosinase Inhibitors.” Journal of Enzyme Inhibition and Medicinal Chemistry 34, no. 1: 279–309. 30734608 10.1080/14756366.2018.1545767 PMC6327992

